# From the Immune Profile to the Immunoscore: Signatures for Improving Postsurgical Prognostic Prediction of Pancreatic Neuroendocrine Tumors

**DOI:** 10.3389/fimmu.2021.654660

**Published:** 2021-04-23

**Authors:** Miaoyan Wei, Jin Xu, Jie Hua, Qingcai Meng, Chen Liang, Jiang Liu, Bo Zhang, Wei Wang, Xianjun Yu, Si Shi

**Affiliations:** ^1^ Department of Pancreatic Surgery, Fudan University Shanghai Cancer Center, Shanghai, China; ^2^ Pancreatic Cancer Multidisciplinary Center, Fudan University Shanghai Cancer Center, Shanghai, China; ^3^ Department of Oncology, Fudan University Shanghai Medical College, Shanghai, China; ^4^ Pancreatic Cancer Institute, Fudan University, Shanghai, China; ^5^ Shanghai Pancreatic Cancer Institute, Shanghai, China

**Keywords:** pancreatic neuroendocrine tumors, Immunoscore, recurrence-free survival, nomogram, prognosis

## Abstract

**Objective:**

Immune infiltration plays an important role in tumor development and progression and shows promising prognostic value in numerous tumors. In this study, we aimed to identify the role of immune infiltration in pancreatic neuroendocrine tumors (Pan-NETs) and to establish an Immunoscore system to improve the prediction of postsurgical recurrence-free survival.

**Methods:**

To derive transcriptional signatures and deconvolute specific immune populations, two GEO datasets containing 158 Pan-NET patients were reanalyzed to summarize the immune infiltration landscape and identify immune-related signatures. Using real-time reverse transcription-polymerase chain reaction, immunofluorescence and immunochemistry methods, candidate signatures were further detected. The least absolute shrinkage and selection operator (LASSO) logistic regression model used statistically significant survival predicators in the training cohort (n=125) to build an Immunoscore system. The prognostic and predictive accuracy was validated in an external independent cohort of 77 patients.

**Results:**

The immune infiltration profile in Pan-NETs showed significant heterogeneity, among which accumulated immune cells, T lymphocytes and macrophages were predominant. Fourteen statistically significant immune-related signatures were further identified in the screening cohort. The Immunoscore system for Pan-NETs (ISpnet) consisting of six immune features (CCL19, IL-16, CD163, IRF4, CD8_PT_ and CD8_IT_) was constructed to classify patients as high and low risk in the training cohort (cutoff value = 2.14). Low-risk patients demonstrated longer 5-year recurrence-free survival (HR, 0.061; 95% CI, 0.026 to 0.14; p < 0.0001), with fewer recurrences and better prognoses. To predict the individual risk of recurrence, a nomogram incorporating both immune signatures and clinicopathological characteristics was developed.

**Conclusion:**

Our model, ISpnet, captures immune feature-associated prognostic indicators in Pan-NETs and represents the first immune feature-based score for the postsurgical prognostic prediction. The nomogram based on the ISpnet and independent clinical risk factors might facilitate decision-making regarding early recurrence risk monitoring, identify high-risk patients in need of adjuvant therapy, and provide auxiliary guidance for patients with Pan-NETs that may benefit from immunotherapy in clinical trials.

## Introduction

Neuroendocrine tumors (NETs) represent heterogeneous malignancies originating from the secretory cells of the diffuse neuroendocrine system and are mainly characterized by indolent growth (1). Gastro-entero-pancreatic neuroendocrine tumors (GEP-NETs) account for most NETs. Specifically, the small intestine (30.8%), rectum (26.3%), colon (17.6%), pancreas (12.1%), and appendix (5.7%) are the most common primary sites in the digestive tract (2), and the incidence has steadily increased in the last 3 decades. Up to 90% of pancreatic neuroendocrine tumors (Pan-NETs) are hormonally silent, and nonfunctioning tumors seem to have a worse prognosis than functioning neoplasms probably because of a late diagnosis. Surgery represents the only curative approach and is recommended to remove all localized and limited metastatic disease. However, 12.3-42% of patients with Pan-NETs experience recurrence after curative resection, and the liver is the most common site (3–5). Currently, various prognostic prediction models have been established mainly based on clinicopathological characteristics, showing inconsistency, and novel molecular profiling markers have also been identified (6–11).

Extensive literature has investigated the host immune response against cancer and demonstrated the prognostic impact of immune infiltration in tumors. A methodology named the ‘Immunoscore’ has been defined to quantify *in situ* immune infiltration. In addition, current studies are consistent with the emerging concept that the neuroendocrine system can be regarded as a subsidiary extension of the innate immune system (12). Notably, the infiltration of immune cells appears to be higher in Pan-NETs than in midgut NETs possibly as a consequence of the higher mutational burden of Pan-NETs (13). A plethora of immune cells, including T cells (14, 15), natural killer (NK) cells (16), macrophages (10, 11, 17, 18), and mast cells (19, 20) have been reported to infiltrate Pan-NETs. Immunomodulatory factors were recently identified as master regulators of GEP-NET metastatic progression and may play a key role in promoting tumor immune escape (21).

In this study, we obtained two RNA-Seq datasets from Pan-NET patients and analyzed the immune characteristics to determine the immune profile of this heterogeneous tumor. Then, we quantified the expression of 10 immune markers (IL-16, IRF4, LRG1, MUC1, CXCL9, CCL19, CR2, PIR, CD79A and TCF21) *via* immunochemistry and CD4, CD8, CD163 distribution intratumorally and peritumorally based on immunofluorescence. The least absolute shrinkage and selection operator (LASSO) Cox regression model, a popular method for regression of high-dimensional predictors (22–24), was applied to establish a novel Immunoscore of Pan-NETs (ISpnet) for survival analysis of patients. Moreover, for clinical use, we constructed a nomogram-derived prognosis system that integrated the ISpnet index with clinicopathological risk factors for the early predictive identification of Pan-NET patients who might experience disease recurrence after surgery.

## Material and Methods

### Dataset Sources, Differential Expression Analysis of Immune Cell Types and Signatures

This study is intended to describe the immune infiltration profile of Pan-NETs. Therefore, two datasets (GSE98894 and GSE73338) from the Gene Expression Omnibus (GEO) were used. The gene expression profile data of primary tumors were used to quantify the infiltration of immune cells in tumor tissues by ssGSEA (single-sample gene set enrichment analysis). Differential gene expression analysis of high and low immune infiltration conditions was performed with the R package “DESeq2”. GSEA enrichment analysis and enrichment map analysis were performed using the ClusterProfiler package. The identification of 150 significant immune signatures was performed using GO:0002376 (immune system process, Gene Ontology Category), which contains 2,776 immune genes to screen candidate genes and verified by qPCR analysis in the Pan-NET patient cohort of our center with 60 patients. The details of the data processing are shown in the **Supplementary Method**.

### Study Population, Clinical Information and Survival Analysis

**Figure 1:**
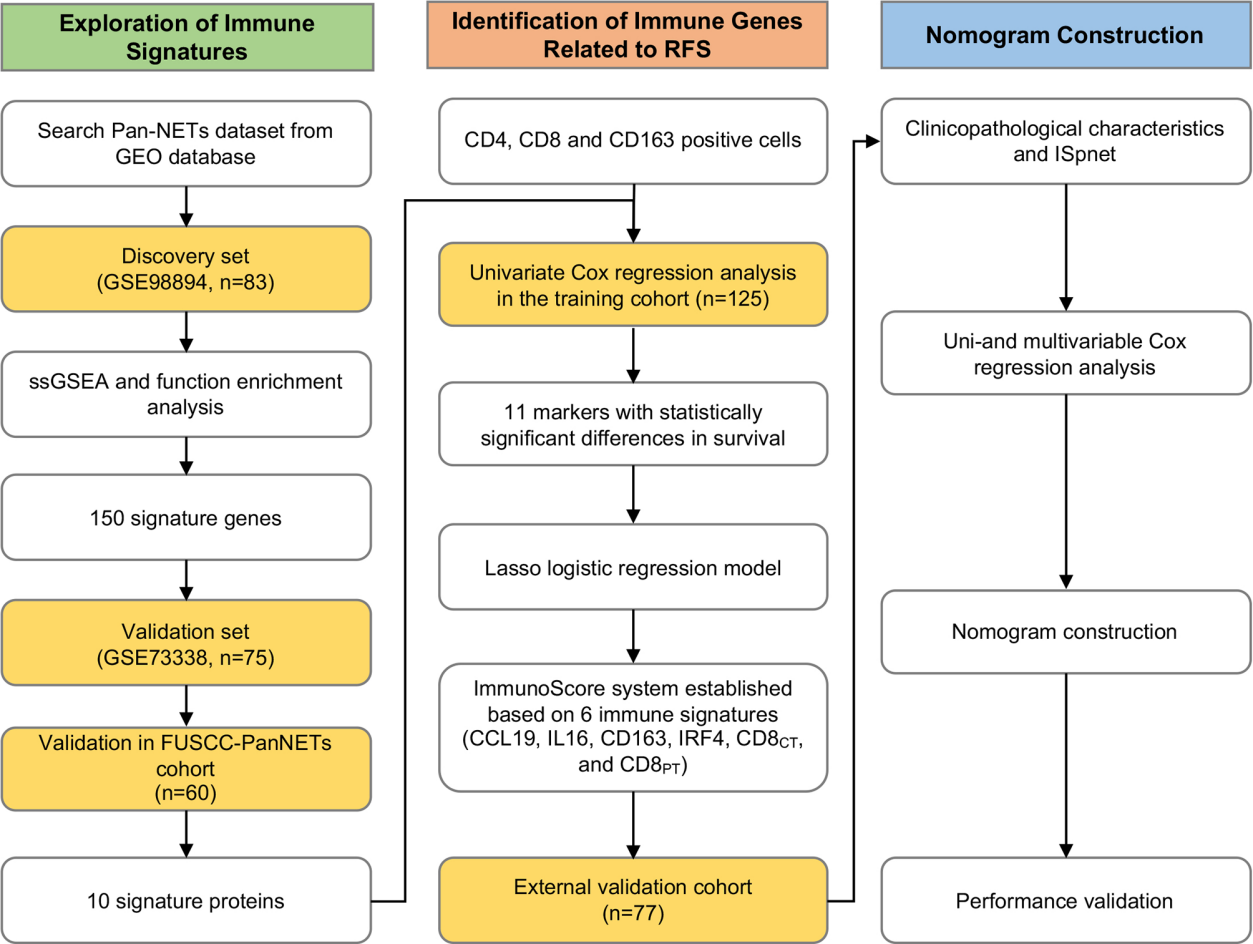
Workflow of the present study.

The study workflow is shown in **Figure 1**. For the training cohort, we used a tissue microarray (TMA) of formalin-fixed, paraffin-embedded (FFPE) specimens from 125 consecutive patients who underwent surgery between March 2012 and May 2018 at the Department of Pancreatic Surgery, Fudan University Shanghai Cancer Center (FUSCC) and were pathologically diagnosed with Pan-NETs that were histologically confirmed, including patients diagnosed with Pan-NET G3 (well differentiated in histology, with Ki-67 over 20%). For each core (1.5 mm in diameter), at least two cores were used for every patient, including the tumor site and adjacent paratumor tissue with histologically normal pancreatic tissues. The validation cohort set comprised 77 patients with Pan-NETs who underwent pancreatectomy at an external hospital with pathology consultations performed at our institute. All clinical information was obtained from medical records, and patients were followed up regularly. Pathology records and site selection in TMA were reviewed and circled by the Department of Pathology at FUSCC. For the survival analysis, we used recurrence-free survival (RFS).

### Reverse Transcription-Quantitative Polymerase Chain Reaction (RT-qPCR), Immunohistochemistry (IHC) and Immunofluorescence (IF) Analysis

Based on the analysis of datasets, the expression of 150 genes was measured by quantitative PCR on sixty frozen Pan-NET tissue samples from patients who had undergone surgery between October 2018 and December 2019 in Shanghai Cancer Center as described in the **Supplementary File**. Furthermore, 10 candidate proteins for IHC staining were identified: IL-16, IRF4, LRG1, MUC1, CXCL9, CCL19, CR2, PIGR, CD79A and TCF21. IHC staining was performed and scored using TMA to determine the protein expression profiles. For calculation and evaluation, the expression levels were based on the score obtained by the intensity of the IHC staining. Briefly, a score was determined according to the intensity of the stain (negative=0, weak=1, intermediate=2, and strong=3). The distribution and numbers of tumor-infiltrating CD4^+^ and CD8^+^ T cells, as well as tumor-associated macrophages (TAMs) (CD163^+^), were determined by IF staining in the TMA and scored in five high-power fields (HPFs) (×400) of a maximal concentration of cells. CD4- and CD8-positive cells are green and red, respectively, according to the fluorescent labels used. The results were evaluated by 2 independent pathologists who were blinded to the clinical outcome. IHC and IF analysis using TMA were performed in the training and validation cohort. The primer sequences and antibodies used in qPCR, IHC and IF staining are listed in **Supplementary Tables S1, S2**.

### Construction of the ISpnet Using the LASSO Cox Regression Model

Univariate analysis using Cox proportional hazards regression modeling was then used to test the significance of different immune markers in this original training group. The p-value for significant markers (< 0.05) remained for further validation within the training group. Specific features were preliminarily selected according to Cox regression in the Pan-NET training cohort of 125 samples from FUSCC, and LASSO regression was performed to confirm significant predictive features for predicting RFS using the R package glmnet. The optimal value of lambda (λ) was tuned *via* ten-fold cross-validation. A score was calculated for each sample *via* a linear combination of the selected features. To determine the optimal cutoff point for the ISpnet value, the survminer package was used, and the minimum group needed to account for at least 20% of the entire cohort. The potential association of the selected feature signature was assessed in the training cohort and then preliminarily validated in the validation cohort by using a calibration plot.

### Construction of an Immune-Based Nomogram Prognostic Model Integrated With Clinicopathological Factors

The ISpnet and patient-specific clinicopathological factors from 125 Pan-NET patients with survival information were subjected to subsequent analyses *via* univariate and multivariate Cox regression. For the multivariable Cox regression model, significant coefficients were used to construct nomograms. The calibration curves were made by plotting the observed rates against the predicted probabilities of the nomogram. A bootstrapping method was used to calculate the concordance index (C-index). The calibration consistency and predictive accuracy of the nomogram were indicated by the 3-, and 5-year RFS rates.

### Statistical Analysis

Data analysis was carried out using SPSS software version 20 (IBM Corporation, New York, USA), GraphPad Prism 8 (GraphPad Software, Inc., La Jolla, USA), R (version 3.6.0; R Foundation for Statistical Computing, Vienna, Austria; https://www.r-project.org/) and RStudio (Version 1.2.1335; RStudio, Inc., Boston, MA; https://www.rstudio.com/). Significance was determined using a one-tailed or two-tailed paired Student’s t test or the Mann-Whitney test as appropriate. The Kaplan-Meier method was used to estimate RFS, and comparisons between curves were performed using the log-rank test. The Cox proportional hazard regression model was used to estimate the hazard ratio (HR) with a 95% confidence interval (CI) for variables associated with RFS. Potential risk factors with a *P* value < 0.05 in the univariate Cox analysis were entered into a multivariate Cox regression model after considering collinearity among variables. The prognostic accuracy of the final model was estimated using time-dependent receiver operating characteristic (ROC) analysis. The area under the ROC curve (AUC) at different cutoff times was measured as prognostic accuracy. Calibration was assessed by visual examination of the calibration plot.

## Results

### Immune Profile and Significant Immune Signatures of Pan-NETs

**Figure 2:**
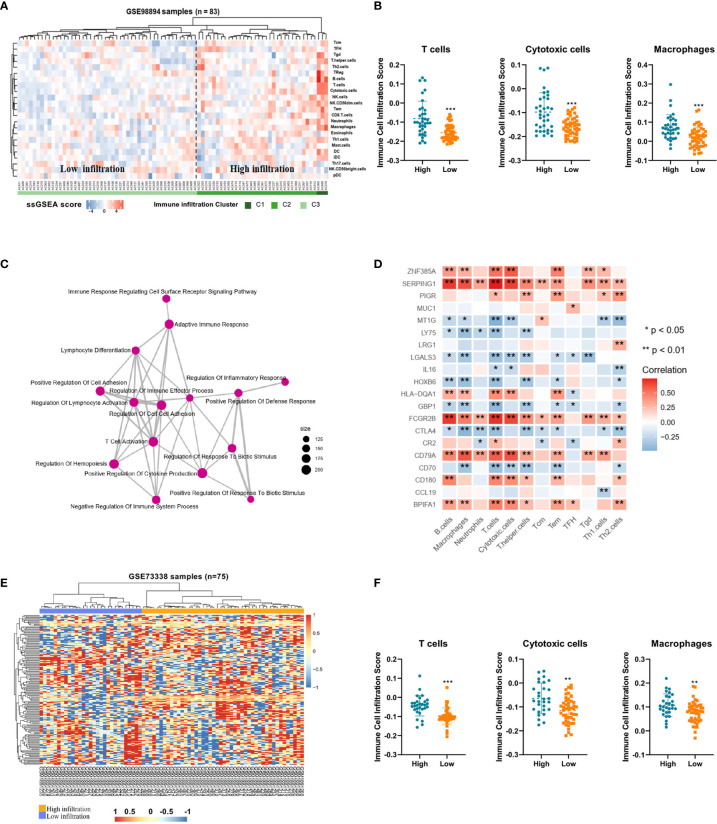
Immune profiles and candidate immune signatures in Pan-NETs revealed by two GSE datasets **(A)** Heatmap for immune cell infiltration from the GSE98894 dataset. **(B)** Enriched T lymphocytes, cytotoxic cells and macrophages showed statistically significant differences between the high- (including high- and moderate-infiltration) and low-infiltration populations. **(C)** GSEA confirmed most of the biological processes of enriched immune signals. **(D)** The correlation of immune signature genes and immune cell types is shown. Red boxes indicate a positive correlation; blue boxes indicate a negative correlation. **(E)** Heatmap for clustering of the GSE73338 dataset with 150 immune signatures resulting in two subgroups with similar immune cell infiltration differences. **(F)** Enriched immune cell types were significantly different in the GSE73338 population. (*indicates 0.05, **indicates 0.01, ***indicates 0.001).

To demonstrate the immune profiles, we assessed the spectrum of immune cell infiltration in Pan-NETs, and the ssGSEA approach was utilized to deconvolve the relative abundance of each cell type based on expression profiling data retrieved from the GSE98894 datasets. Using unsupervised clustering, we categorized the cohort into two subgroups, high- and low-infiltration groups, as shown in **Figure 2A**. The immune cell profiles could be enriched from 24 immune cell types between these two populations with significant differences. The results of GSE98894 datasets were compared and showed that T lymphocytes, cytotoxic cells and macrophages had statistically significant differences (**Figure 2B**, **Supplementary Table S3**). Then, we conducted GSEA and confirmed that most of the biological processes of enriched immune signals were in the process of activation or response (**Supplementary Figure S1**). In **Figure 2C**, the enrichment map identified that T cell activation was the hub functional module, which was consistent with the enrichment of T lymphocytes in the high infiltration group.

**Figure 3:**
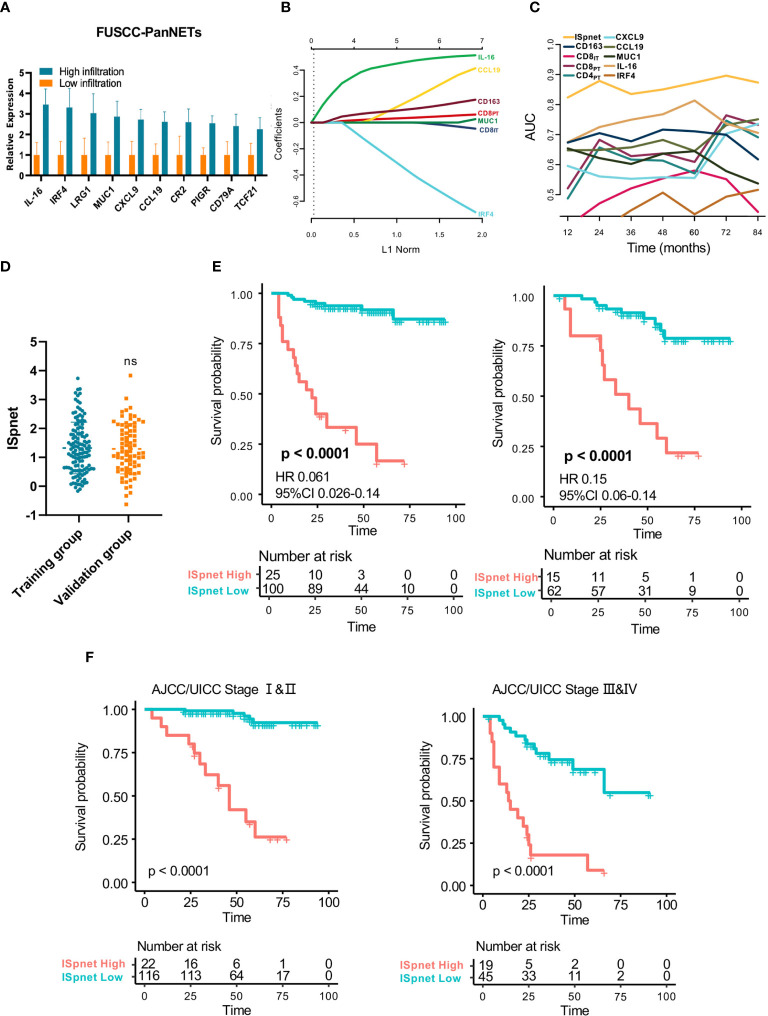
Quantitative Immunoscore establishment and validation in patients with Pan-NETs. **(A)** Ten immune signatures with statistically significant expression differences selected by qPCR methods in 60 Pan-NET patients for further analysis. **(B)** Feature selection using the LASSO regression model. Coefficient profile of the immune-related signatures associated with RFS of patients with Pan-NETs. **(C)** Time-dependent ROC curve describing the prognostic accuracy of the ISpnet and single immune features in the training cohort. **(D)** Kaplan-Meier curves for recurrence-free survival between the immune signature-defined high-risk and low-risk groups in the training and validation cohorts. **(E)** Scatter diagram illustrating the ISpnet of the training and validation cohorts. **(F)** Survival probability of Pan-NET patients with different AJCC/UICC staging system staging in low- and high-ISpnet patients. Ns, No significant.

A total of 150 candidate immune signature genes were selected that met the following criteria: highly correlated with T cell infiltration, ranked according to fold change and an adjusted *P* value *<* 0.05 (**Figure 2D**, **Supplementary Table S4**). Unsupervised clustering of the GSE73338 dataset with 150 candidate genes also resulted in two subgroups with similar immune cell infiltration differences, as shown in **Figure 2E**. The results of GSE73338 datasets also showed that T lymphocytes, cytotoxic cells, and macrophages had statistically significant differences (**Figure 2F**). To further verify the reliability of the expression of 150 immune-related genes, we determined a group of Pan-NET specimens (n=60) with different T cell infiltration profiles and divided them into high and low groups (n=30) for PCR analysis of 150 genes. We found that IL-16, IRF4, LRG1, MUC1 and CXCL9 had the most differential expression changes (**Supplementary Table S5**, **Figure 3A**).

### Clinical Characteristics and Survival Analysis of Pan-NET Patients

The baseline characteristics of the resected patients with Pan-NETs in the training cohort (n=125) and validation cohort (n=77) are summarized in **Table 1**. The average age of the patients at the time of surgery was 51.8 years (range: 25-77); 92 (45.5%) patients were male. The majority (185, 91.6%) of Pan-NETs were nonfunctional. Eighty-three (41.1%) tumors were WHO grade 1, and the remaining 119 (58.9%) were grade 2 and grade 3. The median tumor size was 31 mm (range: 6 to 140 mm). Lymph node (LN) metastasis occurred in approximately one-third of patients. Twenty-eight patients (13.9%) had synchronous liver metastasis at the time of diagnosis and underwent simultaneous liver resection (25 of 28) or intraoperative curative radiofrequency ablation therapy (3 of 21). The median follow-up time was 41 months (IQR 27.00-59.75). Recurrence occurred in 26 (20.8%) patients, including 20 (16%) who developed liver metastasis and 6 (4.8%) with locoregional recurrence.

**Table 1:** Clinical characteristics of patients in the training and external validation cohorts.

	Training cohort (N=125)				Validation cohort (N=77)			
	Patients (n)	high risk	low risk	p	Patients (n)	high risk	low risk	p
N	125	28	97		77	13	64	
Age at surgery (years)								
≤ 55	67 (53.6)	15 (53.6)	52 (53.6)	1	41 (53.2)	6 (46.2)	35 (54.7)	0.797
>55	58 (46.4)	13 (46.4)	45 (46.4)		36 (46.8)	7 (53.8)	29 (45.3)	
Sex								
Female	70 (56.0)	14 (50.0)	56 (57.7)	0.61	40 (51.9)	7 (53.8)	33 (51.6)	1
Male	55 (44.0)	14 (50.0)	41 (42.3)		37 (48.1)	6 (46.2)	31 (48.4)	
Location								
Body&Tail	77 (61.6)	19 (67.9)	58 (59.8)	0.669	47 (61.0)	10 (76.9)	37 (57.8)	0.42
Head	47 (37.6)	9 (32.1)	38 (39.2)		29 (37.7)	3 (23.1)	26 (40.6)	
Multifocal	1 (0.8)	0 (0.0)	1 (1.0)		1 (1.3)	0 (0.0)	1 (1.6)	
Tumor size (cm)								
< 2	25 (20.0)	0 (0.0)	25 (25.8)	<0.001	18 (23.4)	0 (0.0)	18 (28.1)	0.077
2~4	62 (49.6)	12 (42.9)	50 (51.5)		40 (51.9)	8 (61.5)	32 (50.0)	
> 4	38 (30.4)	16 (57.1)	22 (22.7)		19 (24.7)	5 (38.5)	14 (21.9)	
Perineural invasion (PNI)								
Negative	94 (75.2)	19 (67.9)	75 (77.3)	0.44	58 (75.3)	9 (69.2)	49 (76.6)	0.837
Positive	31 (24.8)	9 (32.1)	22 (22.7)		19 (24.7)	4 (30.8)	15 (23.4)	
Lymphovascular invasion (LVI)								
Negative	93 (74.4)	19 (67.9)	74 (76.3)	0.513	58 (75.3)	8 (61.5)	50 (78.1)	0.362
Positive	32 (25.6)	9 (32.1)	23 (23.7)		19 (24.7)	5 (38.5)	14 (21.9)	
AJCC/UICC T stage								
T1	32 (25.6)	2 (7.1)	30 (30.9)	0.004	22 (28.6)	1 (7.7)	21 (32.8)	0.258
T2	53 (42.4)	10 (35.7)	43 (44.3)		35 (45.5)	7 (53.8)	28 (43.8)	
T3	39 (31.2)	16 (57.1)	23 (23.7)		19 (24.7)	5 (38.5)	14 (21.9)	
T4	1 (0.8)	0 (0.0)	1 (1.0)		1 (1.3)	0 (0.0)	1 (1.6)	
AJCC/UICC N stage								
N0	87(69.6)	14(56.0)	73(73.0)	0.056	51(66.2)	9(60.0)	42(67.7)	0.297
N1	33(26.4)	11(44.0)	22(22.0)		21(27.3)	6(40.0)	15(24.2)	
Nx	5(4.0)	0(0.0)	5(5.0)		5(6.5)	0(0.0)	5(8.1)	
AJCC/UICC M stage								
M0	104 (83.2)	21 (75.0)	83 (85.6)	0.303	70 (90.9)	11 (84.6)	59 (92.2)	0.736
M1	21 (16.8)	7 (25.0)	14 (14.4)		7 (9.1)	2 (15.4)	5 (7.8)	
WHO grade								
G1	49 (39.2)	8 (28.6)	41 (42.3)	0.4	34 (44.2)	6 (46.2)	28 (43.8)	0.651
G2	71 (56.8)	19 (67.9)	52 (53.6)		39 (50.6)	7 (53.8)	32 (50.0)	
G3	5 (4.0)	1 (3.6)	4 (4.1)		4 (5.2)	0 (0.0)	4 (6.2)	
Functional								
No	115 (92.0)	27 (96.4)	88 (90.7)	0.558	70 (90.9)	12 (92.3)	58 (90.6)	1
Yes	10 (8.0)	1 (3.6)	9 (9.3)		7 (9.1)	1 (7.7)	6 (9.4)	
CgA								
Negative	5 (4.0)	1 (3.6)	4 (4.1)	1	5 (6.5)	1 (7.7)	4 (6.2)	1
Positive	120 (96.0)	27 (96.4)	93 (95.9)		72 (93.5)	12 (92.3)	60 (93.8)	
Syn								
Negative	1 (0.8)	1 (3.6)	0 (0.0)	0.506	0 (0.0)	0 (0.0)	0 (0.0)	NA
Positive	124 (99.2)	27 (96.4)	97 (100.0)		77 (100.0)	13 (100.0)	64 (100.0)	
Ki67								
1~5%	91 (72.8)	17 (60.7)	74 (76.3)	0.164	19 (24.7)	10 (76.9)	48 (75.0)	1
> 5%	34 (27.2)	11 (39.3)	23 (23.7)		58 (75.3)	3 (23.1)	16 (25.0)	
AJCC/UICC stage								
I	30 (24.0)	1 (3.6)	29 (30.0)	0.026	19 (24.7)	1 (7.7)	21 (32.8)	0.307
II	53 (42.4)	13 (46.4)	40 (41.2)		31 (40.2)	7 (53.8)	26 (40.6)	
III	21 (16.8)	7 (25.0)	14 (14.4)		12 (15.6)	3 (23.1)	12 (18.8)	
IV	21 (16.8)	7 (25.0)	14 (14.4)		15 (19.5)	2 (15.4)	5 (7.8)	

### Screening Results in the Training Cohort With Pan-NETs

To identify the predictive value of immune-related signatures for prognosis, we then selected 10 candidate proteins with statistically significant differences in our FUSCC-PanNET cohort for IHC analysis: CCL19, CXCL9, IL-16, IRF4, MUC1, LRG1, PIGR, CD79A, TCF21, and CR2. Based on our analysis results and previous research (11), TAMs and CD4/8-positive T cells, which are regarded as significant indicators, were also reviewed. Based on the numeration of lymphocyte populations in both the tumor core (IT, intratumoral region) and peritumoral (PT) regions, the mean number of positive cells per HPF indicating the prevalence of immune infiltrates was calculated. Intratumoral TAMs were rarely detected in the Pan-NETs tested. As a result, the total number of TAMs (PT and IT) was used. The median number of peritumoral CD4^+^ T cells/HPF (13.8, range: 0.8-68.8) was significantly higher than that of intratumoral CD4^+^ T cells/HPF (4.8, range: 0-50.7; *P<* 0.0001). The median numbers of peri- and intratumoral CD8^+^ cells/HPF were 7.5 (range: 0.8-72.0) and 6.6 (range: 0.2-44.0), respectively, while there were no differences in CD8^+^ T cell infiltration distribution (*P* > 0.05). The median number of CD163^+^ cells/HPF was 6.6 (range: 0.1-12.0).

In the Cox regression analysis, the cohort was divided according to the number of CD4-, CD8- and CD163-positive cells: high versus medium and low group. For the 10 IHC immune markers, we divided negative and weak staining into the low expression group and intermediate and strong staining into the high expression group. The above nine significant immune markers (CXCL9, CCL19, MUC1, IL-16, IRF4, CD163, CD8_IT_, CD8_PT_, and CD4_PT_) were adopted for further verification using the training cohort of 125 patients according the Cox regression analysis (**Table 2**). The nine selected immune markers showed distinguishable and clear staining as shown in online **Supplementary Figure S2**.

**Table 2:** Univariate Cox regression analysis of prognostic factors in the training cohort.

	Beta	HR (95% CI)	Wald test	p value
IL-16	-1.3	0.28 (0.13-0.6)	10	0.001
CCL19	-0.98	0.37 (0.17-0.81)	6.3	0.012
IRF4	1.3	3.6 (1.2-10)	5.5	0.019
MUC1	-0.87	0.42 (0.19-0.91)	4.9	0.028
CXCL9	-0.87	0.42 (0.19-0.94)	4.4	0.036
TCF21	-0.34	0.71 (0.33-1.5)	0.75	0.390
PIGR	0.026	1 (0.47-2.3)	0	0.950
CD79A	0.81	2.3 (0.98-5.2)	3.6	0.057
LRG1	0.84	2.3 (0.87-6.2)	2.8	0.091
CR2	-0.48	0.62 (0.28-1.3)	1.5	0.220
CD4_CT_	0.45	1.6 (0.63-3.9)	0.94	0.330
CD4_PT_	-0.78	0.46 (0.21-0.99)	4	0.047
CD8_IT_	1.4	3.9 (1.2-13)	5	0.026
CD8_PT_	-0.82	0.44 (0.2-0.95)	4.4	0.036
CD163	-1.7	0.18 (0.08-0.42)	16	<0.001

### Association of the Immune Signatures With Prognosis

The least absolute shrinkage and selection operator (LASSO) logistic regression model was used to establish the Immunoscore system, which involved six markers (percentage of CCL19, IL-16, CD163, IRF4, CD8_IT_, and CD8_PT_) identified by the training set (n=125) (**Figure 3B**, **Supplementary Figure S3A**). The immune signature of each patient was calculated based on their regression coefficients of the expression levels: ISpnet = (0.261 × the status of CCL19) + (0.490 × the status of IL-16) + (0.123 × the status of CD163) + (0.044 × the status of CD8_PT_) – (0.011× the status of CD8_IT_) – (0.493× the status of IRF4). The status of proteins, including CCL19, IL-16 and IRF4, is the IHC staining score. The status of CD8_PT_, CD8_IT_, and CD163 indicates the mean number of positive cells in five high-power fields (×400) of a maximal concentration of cells. In the training cohort, the patients were separated into low-risk and high-risk groups using an optimal cutoff value (ISpnet=2.14) generated by Survminer. The low-risk and high-risk groups comprised 77.6% (97/125) and 22.4% (28/125) of the patients, respectively. The 5-year RFS was 16.7% (95% CI, 5.29% to 52.6%) in the high-risk group and 91.7% (95% CI, 95.9% to 98.1%) in the low-risk group (HR, 0.061; 95% CI, 85,8% to 98.1% 0.026 to 0.14; p<0.0001). The ROC curve described the 5-year prognostic accuracy of the ISpnet and single immune features in the training cohort (**Figure 3C**, **Supplementary Figure S3B**).

Given the better accessibility and similar prognostic value of the immune signature ISpnet, it was selected to predict the prognosis of patients with Pan-NETs in the validation cohort. A scatter diagram illustrating the immune signature of the training and validation cohorts is shown in **Figure 3D**, with no significant difference in immune signature distribution observed between the two groups (p=0.998). In the validation cohort, ISpnet categorized 62 (80.5%) of the 77 patients into the low-risk group and 15 patients (19.5%) into the high-risk group. Patients in the high-risk group demonstrated far shorter RFS (HR, 0.15; 95% CI, 0.06 to 0.14; p<0.0001; **Figure 3E**) than patients in the low-risk group. We also performed stratified analyses of Pan-NET patients with stage I-II and stage III-IV disease in the whole cohort according to the 8^th^ edition AJCC/UICC TNM staging system. Low-ISpnet patients had longer RFS than high-ISpnet patients (*P<* 0.0001, **Figure 3F**).

### Establishment and Validation of a Nomogram With the Immune Signatures

Univariate and multivariate Cox regression analyses were conducted to explore whether the prognostic value of the ISpnet was independent of conventional clinicopathological characteristics in our cohort with Pan-NETs (**Table 3**). After multivariable adjustment by clinicopathological risk factors, the results confirmed the robustness of ISpnet for independently predicting RFS in the training cohort (HR, 0.091; 95% CI, 0.035 to 0.23; p < 0.0001) and in the validation cohort (HR, 0.13; 95% CI, 0.04 to 0.38; p < 0.001). In addition, WHO grade (HR, 9.220; 95% CI, 1.120 to 75.908; p = 0.039), and liver metastasis (HR, 3.879; 95% CI, 1.581 to 9.519; p = 0.003) also remained significant for RFS after adjustment for various cofactors.

**Table 3:** Univariate and multivariate Cox regression analysis of prognostic factors in the training and validation cohorts.

Training cohort	Univariate		Multivariate	
	HR (95% CI)	p	HR (95% CI)	p
T category (T3-4 vs T1-2)	6.9 (2.9-16)	<0.001	1.9 (0.7-5.2)	0.199
N category (N1 vs N0)	5.8 (2.6-13)	<0.001	1.6 (0.7-3.9)	0.303
Liver metastasis (M1 vs M0)	9.8 (4.3-22)	<0.001	3.9 (1.6-9.5)	0.003
WHO grade (G2-3 vs G1)	22 (2.9-160)	0.003	9.2 (1.1-75.9)	0.039
ISpnet (low vs high)	0.061 (0.026-0.140)	<0.001	0.091 (0.035-0.235)	<0.001
**Validation cohort**				
	HR (95% CI)	p	HR (95% CI)	p
T category (T3-4 vs T1-2)	2.9 (1.2-6.9)	0.019	1.5 (0.6-4.1)	0.379
N category (N1 vs N0)	3.2 (1.3-8.1)	0.011	2.3 (0.7-7.2)	0.156
Liver metastasis (M1 vs M0)	9.4 (3.3-26)	<0.001	9.5 (2.6-34.7)	<0.001
WHO grade (G2-3 vs G1)	9.6 (2.2-42)	0.003	6.3 (1.3-29.8)	0.021
ISpnet (low vs high)	0.15 (0.06-0.35)	<0.001	0.132 (0.045-0.389)	<0.001

**Figure 4:**
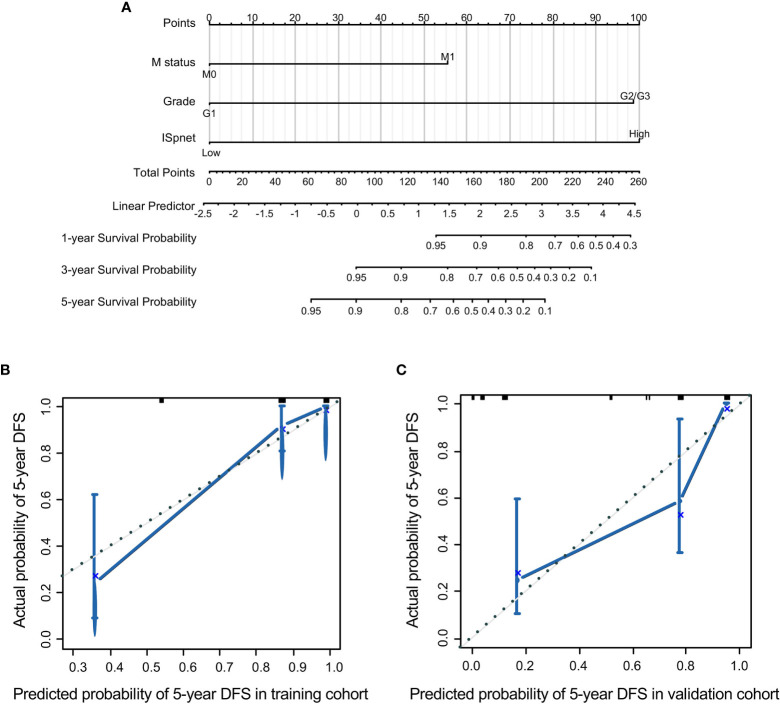
Nomogram for predicting the RFS of Pan-NET patients. **(A)** Nomogram scoring system based on the independent predictors from the multivariable Cox regression analysis to predict the risk of RFS in Pan-NETs. **(B, C)** Calibration plots of the nomogram to predict RFS at 5 years in the training cohort and the validation cohort, indicating good agreement between the prediction and the observation.

Nomograms, with the ability to generate an individual probability of a clinical event by integrating diverse prognostic and determinant variables, are widely used as prognostic devices in oncology and medicine. Thus, a nomogram to predict the 3- and 5-year RFS was made that incorporated the ISpnet, synchronous liver metastasis, and grade (**Figure 4A**). In this nomogram, the recurrence score predicts the probability of developing recurrence after resection in patients with Pan-NETs. Among them, ISpnet had the highest C-index (0.796; 95% CI, 0.714 to 0.878; C-index, 0.714; 95% CI, 0.607 to 0.821, respectively) in both the training and validation cohorts. Liver metastasis and grade contributed to the most risk points after ISpnet. The calibration plots for the nomogram indicated good agreement in the training cohort (C-index, 0.917; 95% CI 0.884 to 0.950) and the validation cohort (C-index, 0.864; 95% CI, 0.798 to 0.930) (**Figures 4B, C**).

We also compared the predictive accuracy of this nomogram with individual predictors in the validation cohort; the nomogram performance (C-index, 0.864) was better than that of the ISpnet (C-index, 0.714), liver metastasis (C-index, 0.673), and grade (C-index, 0.676) (**Supplementary Table S6**). In summary, these findings suggest that the nomogram is a better model for predicting RFS in patients with Pan-NETs.

## Discussion

Since the early 1900s, immune infiltration of cancers has been believed to be a positive factor for patient outcome, and immunotherapy has recently modified cancer treatment (25, 26). Efforts are currently underway to explore the efficacy of immune checkpoint inhibitors in patients with Pan-NETs (27, 28). However, these insights have not had a major influence on cancer classification or clinical decision-making, and the immune cell landscape in patients with Pan-NETs has not yet been explored or summarized thoroughly.

Here we utilized GSE98894 and GSE73338 combined with the immune cell list provided by Bindea et al. (29) to calculate the Immunoscore and divided 158 patients into high- and low-infiltration types, which demonstrated that Pan-NETs manifested with diverse immune infiltration, underlying the importance of immunologic biomarkers in predicting prognosis and the response to therapy. Through biological analysis, we found that T lymphocyte-oriented immune cells exhibit highly infiltrating characteristics in patients with Pan-NETs, yielding the same results proposed by Cai et al. (11). Low peritumoral CD4^+^ cell infiltration, high intratumoral CD8^+^ T cell infiltration, and low peritumoral CD8^+^ T cell infiltration were significantly associated with RFS in Pan-NET patients, partly in accordance with the conclusions reached by other researchers (11). It is well known that T lymphocytes are at the center of inducing an effective adaptive immune response and maintaining homeostasis and that the degree of T cell infiltration of tumors has been considered both a general prognostic factor and a specific predictor of the response to checkpoint inhibition (13). Inspired by this knowledge, our study further explored and revealed novel immune signatures in patients with Pan-NETs, including IL-16, CXCL9, CCL19, IRF4, and MUC1 expression profiles, which strengthen the evidence regarding the accumulation of tumor-induced cytokines, chemokines, immune-related regulatory factors, and glycoprotein antigens in Pan-NET tissues (30, 31).

International validation results support the implementation of a consensus Immunoscore as a new component of a TNM-Immune classification of cancer (32, 33). The Immunoscore could have several potential clinical applications, such as prognostic and theranostic applications (22, 34). In carcinoid tumors, especially in lung NETs, the Immunoscore may act as a further prognostic indicator (35). To our knowledge, there is no Immunoscore system for Pan-NETs reported in the literature, and we are the first to introduce this system. In this study, we constructed an immune classifier named the ISpnet comprising 6 immune-related signatures, CD8_PT,_ CD8_IT_, CCL19, IL-16, IRF4 and CD163, as a prognostic tool independent of the AJCC/UICC staging or other clinicopathological factors to predict survival in Pan-NET patients with surgical resection. Specifically, CD8^+^ cytotoxic T lymphocytes are preferred immune cells for targeting cancer and are needed in a process called tumor immunity cycle for making durable and efficient antitumor immune responses (36). The cytokine CCL19 plays an important role in the active recruitment, trafficking and migration of T lymphocytes. IL-16 is associated with macrophages and Th cells (37). IRF4 plays a key role in the expression of T cell genes and the development of the T cell immune response (38). CD163 is a marker of tumor-associated macrophages with an M2 phenotype. Specific depletion of CD163^+^ macrophages results in massive infiltration of activated T cells and tumor regression (39). Pan-NET patients with a low ISpnet demonstrated fewer recurrences and better prognoses than patients with a high ISpnet, and most genes related to liver metastasis exhibited high expression in the high-infiltration population. Thus, intriguingly, progressive activation of the immune system during Pan-NET progression and mutation accumulation was envisaged.

The AJCC/UICC staging classification is crucial in assessing prognosis and establishing a treatment strategy in patients with Pan-NETs but provides limited prognostic information and does not predict the response to therapy. The established ISpnet classifier could indicate the bioimmunological characteristics of the Pan-NET population and contains significant signatures mainly related to T lymphocyte features. This classifier was constructed using LASSO Cox regression models and complements clinicopathological factors that could greatly improve its predictive accuracy. Currently, incorporating the Immunoscore as a prognostic factor and introducing immune parameters into cancer classification and as an integral part of management to guide therapeutic decisions is indicated (32, 35, 40). Thus, a nomogram that integrates the ISpnet with important risk factors indeed provides more comprehensive information than AJCC/UICC staging. More meaningfully, the multi-immune feature-based classifier may help optimize the adjuvant therapy protocol in patients with Pan-NETs. Beyond the results obtained in localized tumors, the relevance of the Immunoscore could extend to metastatic disease because the Immunoscore identifies tumors that are likely to metastasize and predicts the prognosis of patients with metastases from primary Pan-NETs.

A recent study demonstrated the immune microenvironment of Pan-NETs and identified the metastasis-like primary (MLP)-1 subtype as an immune-high phenotype featuring broad and robust activation of immune-related genes. The MLP-1 subtype was first identified by mRNA and miRNA transcriptome profiles and signature genes of PanNET tumors, which were characterized by poorly differentiated tumors associated with liver metastases, high proliferative activity and aggressive behavior (41). The existence of a human MLP-like Pan-NET cluster has also been confirmed by a whole-genome study (42). Based on multiple transcriptome profiling, Young K et al. (43) demonstrated that this subtype contains high levels of lymphocytes and macrophages, which yields similar results proposed by recent literature and our analysis. Both of the studies focused on the immune landscape of Pan-NETs, and an analysis conducted by Young K et al. emphasized the MLP subtypes. We aim to establish a postsurgical prognostic prediction system based on the immune signatures derived from transcriptome datasets, and the Young K et al. tried to identify potential therapeutic vulnerabilities in this disease and pave the way for future precision immunotherapy studies. There maybe a link between our signatures and these subgroups, which need for further investigation in clinical cases.

Our study has some limitations. This was a retrospective study on data obtained from a single institution. There was also a lack of external validation with limited generalizability, as all specimens were obtained from patients at our center. The adequacy of TMA cores compared to whole slides for such an analysis may bring the inevitable discrepancy. Another limitation of our ISpnet is that it was based on T lymphocyte-related signatures and did not include more features, such as CCR7 and CD56. In addition, this is the first version of a possible score that could be adapted with specific antibodies, or could be simplified using less markers with more adapted antibodies, the validation of suitable antibodies on signatures revealed by our result on multicenter patient populations should be necessary before applied it into standard use consideration. The current study had insufficient adjuvant therapy data, which may influence the prognosis and result in limitations to its application. Thus, a prospective study will be needed to further validate our findings.

## Conclusion

Collectively, we established the first Immunoscore for Pan-NETs, the ISpnet, which might be a useful predictive tool to identify patients with different prognoses who might benefit from immunotherapy, especially high-ISpnet patients, who may show a response to T cell checkpoint inhibition. Moreover, the nomogram encompassing the ISpnet and patient-specific clinicopathological characteristics could integrate valuable patient-specific information and effectively predict RFS in Pan-NET patients. Thus, a nomogram based on the ISpnet might facilitate decision-making regarding early recurrence risk monitoring, identify high-risk patients in need of adjuvant therapy, and provide auxiliary guidance for patients with Pan-NETs who may benefit from immunotherapy in clinical trials.

## Data Availability Statement

The original contributions presented in the study are included in the article/**Supplementary Material**. Further inquiries can be directed to the corresponding authors.

## Ethics Statement

The studies involving human participants were reviewed and approved by the Ethics Committee of the Fudan University Shanghai Cancer Center. The patients/participants provided their written informed consent to participate in this study. The animal study was reviewed and approved by the Ethics Committee of the Fudan University Shanghai Cancer Center.

## Author Contributions

MW, SS and XY conceptualized and designed the study. JH, QM, CL, JL and BZ were in charge of data acquisition, MW, JX and WW performed analysis. XY and SS interpreted the data. MW and JX drafted the manuscript. XY and SS revised the manuscript. All authors contributed to the article and approved the submitted version.

## Funding

This study was jointly funded by the National Natural Science Foundation of China (No. 81772555, 81802352 and 81902428), the National Science Foundation for Distinguished Young Scholars of China (No. 81625016), the Shanghai Sailing Program (No. 19YF1409400 and 20YF1409000), the Shanghai Rising-Star Program (No. 20QA1402100), the Shanghai Anticancer Association Young Eagle Program (No.SACA-CY19A06), the Clinical and Scientific Innovation Project of Shanghai Hospital Development Center (No. SHDC12018109 and SHDC12019109) and the Scientific Innovation Project of Shanghai Education Committee (No. 2019-01-07-00-07-E00057).

## Conflict of Interest

The authors declare that the research was conducted in the absence of any commercial or financial relationships that could be construed as a potential conflict of interest.
