# Supplementary material for: From the Immune Profile to the Immunoscore: Signatures for Improving Postsurgical Prognostic Prediction of Pancreatic Neuroendocrine Tumors
Source: Front Immunol. 2021 Apr 23;12:654660. doi: 10.3389/fimmu.2021.654660 (PMC8102869; doi:10.3389/fimmu.2021.654660)
Supplement: Supplementary file 1 [file DataSheet_1.docx]

**Supplementary Material**

**Methods**

**Dataset sources**

Alvarez et al. [1] introduced a new precision oncology framework and identified several master regulator proteins in GEP-NETs, including key regulators of the neuroendocrine lineage progenitor state and immunoevasion, providing transcriptomes profiled by RNA-Seq (accession number: GSE98894) containing primary and liver metastatic Pan-NET samples (83 and 30, respectively). Moreover, Missiaglia et al. [2] investigated the global gene expression in 75 primary Pan-NETs using an 18.5 K human oligo microarray (accession number: GSE73338).

**Implementation of single-sample gene set enrichment analysis (ssGSEA) and identification of immune signatures**

Above two datasets were obtained from the Gene Expression Omnibus (GEO) data repository, and raw count data with EntrezID were downloaded. We transferred raw counts into transcripts per kilobase million (TPM) values with the following formula, which are more comparable between samples, for further analysis.

$${TPM}_{i}=\frac{X_{i}}{\tilde{l_{i}}}\cdot\left( \frac{1}{\sum_{j} \frac{X_{j}}{\tilde{l_{j}}}} \right)\cdot{10}^{6}$$

The infiltration levels of immune cell types were quantified and determined by single-sample gene set enrichment analysis (ssGSEA) in the R package GSVA. In total, we identified 24 immune cells that are involved in innate immunity [NK cells, CD56^dim^ NK cells, CD56^bright^ NK cells, dendritic cells (DCs), plasmacytoid dendritic cells (pDCs), immature DCs (iDCs), activated DCs (aDCs), neutrophils, mast cells, eosinophils, and macrophages] and adaptive immunity [T cells, T helper cells, B cells, CD8 T cells, cytotoxic cells, central memory T (Tcm), effector memory T (Tem), T follicular helper (TFH), Tγδ/Tgd, Th1, Th2, Th17, and Treg cells] [3,4]. Consensus clustering for class discovery based on the comparison of immune profiles using the R package “ComplexHeatmap” was conducted and is presented as heatmaps. Differential expression analysis of immune cell types was performed with the R package “DESeq2”. *P* values were adjusted for multiple testing with Benjamini-Hochberg. GSEA was performed by using the “clusterProfiler” package to impute the functional pathway.

And then we use GO:0002376 (immune system process, Gene Ontology Category) which contains 2, 776 genes to screen candidate genes. First, we used the R package "DESeq2" to perform differential expression analysis on samples in the GSE98894 database. Second, we need to ensure that candidate genes are highly correlated with T cell infiltration. We calculated the correlation between the expression value of 2, 776 genes and the value of T cells infiltration calculated by ssGSEA. Candidate genes need to meet the significant correlation with T cell infiltration, and meanwhile have significant differential expression. In this step, we obtained 88 candidate genes. Third, in view of the fact that immune processes other than T cell activation are also highly enriched, we selected 62 of the remaining genes with the most differential changes to form a list of 150 candidate genes. Finally, in Pan-NET patient cohort of our center with 60 patients, we stained the T cells with CD3 in the tissues and then divided into high-infiltration and low-infiltration groups. We then used qPCR to verify the expression of 150 genes.

**Reverse transcription-quantitative polymerase chain reaction (RT-qPCR), immunohistochemistry (IHC) and immunofluorescence (IF) analysis**

Sixty Pan-NET tissue samples (SHPCI-PanNET cohort) were obtained from patients who had undergone surgery between October 2018 and December 2019 in Shanghai Cancer Center. All cases were reviewed by a pathologist and histologically confirmed and patients recruited received no other treatments prior to surgery. All samples were snap frozen in liquid nitrogen and stored at -80°C prior to RNA isolation. Informed consent was obtained from all patients. Total RNA was extracted from frozen material using TRIZOL (TaKaRa, Dalian, China) according to the manufacturers’ protocol. RNA was reversed transcribed into cDNA using the Primer-Script one step RT-PCR kit (TaKaRa, Dalian, China). The cDNA template was amplified by real-time RT-PCR using the SYBR Premix Dimmer Eraser kit (TaKaRa). The primer sequences used were as follows in Supplementary Table 1. Real-time-PCR reactions were performed by the ABI7500 system (Applied Biosystems, Carlsbad, CA, USA). The qPCR amplification was performed in triplicate reactions beginning at 95°C for 10 min, followed by 40 cycles of 95°C for 10 s, and 60°C for 60 s. Quantitative normalization of candidate genes cDNA was performed in each sample using the expression of the GAPDH as an internal control. The relative level of candidate signature transcripts to control GAPDH was determined by the 2^-ΔΔCT^ method. All experiments were performed in triplicate.

IHC staining of the whole TMA was performed and scored to determine the expression of ten candidate signatures (IL-16, IRF4, LRG1, MUC1, CXCL9, CCL19, CR2, PIGR, CD79A and TCF21). Briefly, after dewaxed, rehydrated and processed for antigen retrieval, endogenous peroxidase was quenched with 3% H_2_O_2_ for 20 min, and then nonspecific reaction was blocked with 5% BSA for 30 min. The slide was incubated overnight with primary antibody at 4℃. After washing, the slide was incubated with HRP-conjugated secondary antibody. Staining was visualized by incubation with DAB for 3 minutes and then examined on a microscope. For calculation and evaluation, the expression levels were based on the score obtained by the intensity of the IHC staining. The intensity of the staining was recorded as 0, 1, 2, and 3, referring to negative, weak, intermediate, and strong staining, respectively.

The preceding procedures of immunofluorescence of CD4 and CD8 positive T lymphocytes, CD163 positive TAM are the same with IHC protocol. The slides were incubated with secondary fluorescence antibody. After washing, adding spontaneous fluorescence quenching reagent to incubate for 5 min, then incubate with DAPI solution. Microscopy detection and collect images by Fluorescent Microscopy, DAPI glows blue by UV excitation wavelength 330-380 nm and emission wavelength 420 nm; FITC glows green by excitation wavelength 465-495 nm and emission wavelength 515-555 nm; CY3 glows red by excitation wavelength 510-560 nm and emission wavelength 590 nm. Nucleus is blue by labeling with DAPI. Staining obtained with CD4, CD8, and CD163-specific monoclonal antibodies (mAbs) was scored by counting the number of stained T lymphocytes and TAMs in five high-power fields (HPF) (×400) of a maximal concentration of cells. Based on the numeration of lymphocyte and TAM populations in both the tumor core (IT, intratumoral) and the peritumoral (PT), the mean number of positive cells per HPF indicating the prevalence of immune infiltrates were calculated. The results were evaluated by 2 independent pathologists who were blinded to the clinical outcome.

**References**

1. Alvarez MJ, Subramaniam PS, Tang LH*, et al.* A precision oncology approach to the pharmacological targeting of mechanistic dependencies in neuroendocrine tumors. Nat Genet 2018;50(7):979-989.

2. Missiaglia E, Dalai I, Barbi S, *et al.* Pancreatic endocrine tumors: expression profiling evidences a role for AKT-mTOR pathway. J Clin Oncol 2010; 28(2):245-55.

3. Zhang L, Zhao Y, Dai Y*, et al.* Immune Landscape of Colorectal Cancer Tumor Microenvironment from Different Primary Tumor Location. Front Immunol 2018;9:1578.

4. Bindea G, Mlecnik B, Tosolini M*, et al.* Spatiotemporal dynamics of intratumoral immune cells reveal the immune landscape in human cancer. Immunity 2013;39(4):782-95.

**Supplementary figure legends**

**Figure S1.** **GSEA showed the enriched pathways in Pan-NETs derived the two datasets.**

**Figure S2.** **Candidate immune signatures in Pan-NETs.**

(A) IHC results revealed high and low expression patterns of 5 candidate immune signatures.

(B) Representative IF staining patterns of CD4^+^, CD8^+^ T lymphocytes in peri- and intratumoral.

(C) Representative IF staining patterns of CD163^+^ TAMs in peri- and intratumoral.

**Figure S3. Quantitative Immunoscore establishment and validation in patients with Pan- NETs.**

(A) Tuning parameter (selection by 10-fold cross-validation via minimum criteria). Partial likelihood deviance was plotted versus log(c).

(B) ROC curve described the 5-year prognostic accuracy of the ISpnet and single immune feature in the training cohort.

| **Gene** | **Forward primer 5’-3’** | **Reverse primer 5’-3’** |
| --- | --- | --- |
| GADPH | GTCAACGGATTTGGTCTGTATT | AGTCTTCTGGGTGGCAGTGAT |
| CCL19 | GCCTGCTGGTTCTCTGGACTTC | GTCTCTGGATGATGCGTTCTACCC |
| CXCL9 | TCTGATTGGAGTTCAAGGAGC | CATGTTTGGTCTCCATTCTTCA |
| IL-16 | GCCGAAGACCCTTGGGTTAG | GCTGGCATTGGGCTGTAGA |
| IRF4 | AGATTCCAGGTGACTCTGTG | CTGCCCTGTCAGAGTATTTC |
| MUC1 | CCATTCCACTCCACTCAGGT55555AGGGCCAGAG | CCACATGAGGCTTCCACACAC55555AGTGTCCGAG |
| LRG1 | TGCCAGCCAACCTCCTCCAG | GTCAGGGCGTTTCGGGTTAGATC |
| PIGR | ACAGCGAGGGGTGGGTTAAGG | ATGACAGTGAAGGTGCCGTTGC |
| CD79A | TCTTCCTCCTCTTCCTGCTGTCTG | CGTTGGCGTTGTTGCTGCTATTG |
| TCF21 | CAGCGATGTGGAGGACCTTCAAG | TCTCCTCGGTGCTCTCGTTGG |
| CR2 | CTACTTCTGCGGTTCAGTGTCCAC | TCGGATTTGCTTGCTGCCCTTC |

Table S1. Primer sequences used in qPCR

Table S2. List of antibodies used for IHC and IF staining

| **Signatures** | **Dilution** | **Source** |
| --- | --- | --- |
| CD79A | 1:150 | Santa Cruz Biotechnology, sc-20064 |
| CR2/CD21 | 1:200 | Abcam, ab75985 |
| TCF21 | 1:150 | Abcam, ab32981 |
| CCL19 | 1:100 | Proteintech,13397-1-AP |
| CXCL9/MIG | 1:100 | Proteintech, 22355-1-AP |
| MUC1 | 1:100 | Proteintech, 19976-1-AP |
| LRG1 | 1:100 | Proteintech, 13224-1-AP |
| PIGR | 1:50 | Proteintech, 22024-1-AP |
| IRF4/MUM1 | 1:50 | Abcam, ab104803 |
| IL-16 | 1:100 | Proteintech-66145-1-AP |
| CD4 | 1:100 | Abcam, ab 133616 |
| CD8  CD163 | 1:100  1:100 | Servicebio, GB13068-2  Servicebio, GB11340-1 |

| Table S3. P value of different immune cell types: high infiltration vs. low infiltration group | | |
| --- | --- | --- |
| **Immune cell types** | **GSE98894** | **GSE73338** |
| NK CD56 dim cells | 0.00000155 | 0.00166568 |
| Neutrophils | 0.00000204 | 0.076171366 |
| T cells | 0.0000173 | 1.49102E-05 |
| Th1 cells | 0.0000441 | 0.38410701 |
| Macrophages | 0.000150653 | 0.010242175 |
| Tem | 0.00020973 | 0.029563872 |
| Cytotoxic cells | 0.000358788 | 0.001489139 |
| Th2 cells | 0.002489493 | 0.036721277 |
| Tcm | 0.00550935 | 0.203056143 |
| B cells | 0.006895135 | 0.157266456 |
| NK cells | 0.011515274 | 0.452845098 |
| Th17 cells | 0.018241682 | 0.102697681 |
| T helper cells | 0.031667421 | 0.281452122 |
| TFH | 0.035511541 | 0.133262333 |
| Tgd | 0.035511541 | 0.24438414 |
| iDC | 0.058706524 | 0.276620526 |
| Eosinophils | 0.067875119 | 0.003084355 |
| DC | 0.072149939 | 0.760652631 |
| CD8 T cells | 0.102640343 | 0.486251523 |
| aDC | 0.264512866 | 0.157266456 |
| TReg | 0.346919279 | 0.810834945 |
| Mast cells | 0.586398846 | 0.065970701 |
| NK CD56 bright cells | 0.832035304 | 0.000423589 |
| pDC | 0.868165327 | 0.072636972 |
|  |  |  |

| Table S4. Differential gene expression analysis of 150 immune gene in GSE98894 dataset | | | | | | |
| --- | --- | --- | --- | --- | --- | --- |
| Symbol | **Base Mean** | **log^2^ FoldChange** | **lfcSE** | **stat** | **P value** | **padj** |
| TRIM29 | 15.81894972 | -2.460571378 | 0.643173303 | -3.825673991 | 0.000130415 | 0.010456054 |
| LAX1 | 26.36240163 | -0.250947443 | 0.284680959 | -0.881504136 | 0.378045016 | 0.812550768 |
| CCR4 | 9.160882818 | -0.324180808 | 0.43636858 | -0.742905935 | 0.457538633 | 0.849855861 |
| MZB1 | 20.83032822 | -1.046143394 | 0.387726993 | -2.698144345 | 0.00697272 | 0.138571923 |
| PIGR | 478.4694518 | -3.340852422 | 0.614030384 | -5.44085848 | 5.30244E-08 | 2.37362E-05 |
| CD70 | 3.232028452 | -0.149494652 | 0.430526659 | -0.347236691 | 0.728413508 | 0.939663747 |
| HOXA3 | 177.5343792 | -0.439345405 | 0.336036152 | -1.307434934 | 0.191065039 | 0.655029302 |
| IL16 | 413.901797 | -0.35421871 | 0.222428702 | -1.592504507 | 0.111271391 | 0.532238381 |
| IL6 | 82.60805706 | -1.323038678 | 0.448594464 | -2.949297829 | 0.003184969 | 0.086957338 |
| TIGIT | 40.75269897 | -0.538939224 | 0.369979479 | -1.456673287 | 0.145206587 | 0.589913771 |
| CCL19 | 99.36230989 | -1.068493173 | 0.503263115 | -2.123130306 | 0.033742934 | 0.319962235 |
| SELE | 102.5704091 | -0.509963819 | 0.417578048 | -1.221241926 | 0.221994435 | 0.689205955 |
| THEMIS | 28.84192051 | -0.100658997 | 0.321960156 | -0.312644267 | 0.754550943 | 0.946539116 |
| CXCL9 | 179.0421016 | -1.391793489 | 0.412846066 | -3.371216544 | 0.00074837 | 0.03420462 |
| CTLA4 | 27.16878618 | -0.638900531 | 0.354232951 | -1.803616884 | 0.071291387 | 0.443368463 |
| IRF4 | 38.51362915 | -0.814014514 | 0.396264192 | -2.05422173 | 0.039954236 | 0.34747672 |
| ZAP70 | 138.9471506 | -1.184746351 | 0.328882291 | -3.602341573 | 0.000315364 | 0.019328621 |
| MUC1 | 591.762031 | -1.156881995 | 0.398444629 | -2.903495016 | 0.003690228 | 0.096149991 |
| REG1B | 301.9095616 | -7.843985554 | 1.391074287 | -5.638797028 | 1.71242E-08 | 9.1987E-06 |
| CXCL11 | 24.64349582 | -2.069231251 | 0.52805796 | -3.918568429 | 8.90764E-05 | 0.00797494 |
| LGALS3 | 1134.27837 | 0.03202663 | 0.342674948 | 0.093460669 | 0.925537594 | 0.984777373 |
| SIRPG | 21.39989573 | -0.621037758 | 0.333250668 | -1.863575435 | 0.06238135 | 0.420376155 |
| SIT1 | 12.80720261 | -0.740036985 | 0.377688597 | -1.959383979 | 0.050067835 | 0.379066722 |
| CNR1 | 266.7851682 | -1.1684984 | 0.422079132 | -2.768434427 | 0.005632632 | 0.121881533 |
| AIM2 | 25.82000149 | -1.530034077 | 0.423347691 | -3.614131149 | 0.000301356 | 0.018823393 |
| MYO10 | 6968.299823 | -0.096775279 | 0.289597429 | -0.334171749 | 0.738249961 | 0.942329239 |
| TNFRSF1B | 667.4685313 | -0.43758575 | 0.229664696 | -1.905324401 | 0.056737915 | 0.401457572 |
| LRG1 | 161.7173735 | -0.157727909 | 0.539527352 | -0.292344602 | 0.77002316 | 0.951563487 |
| CD79A | 26.18739293 | -2.131414111 | 0.415768507 | -5.126444347 | 2.95265E-07 | 9.61267E-05 |
| CRTAM | 10.43552018 | -0.733541278 | 0.377209972 | -1.944649749 | 0.051817147 | 0.385791769 |
| TCF21 | 81.50997833 | -0.255527069 | 0.319451392 | -0.799893428 | 0.423772546 | 0.835398197 |
| IL2RG | 176.9409762 | -1.114024039 | 0.320046589 | -3.48081835 | 0.000499884 | 0.026261652 |
| CCL5 | 177.6103329 | -0.877031204 | 0.293500257 | -2.988178654 | 0.002806455 | 0.079188331 |
| CR2 | 21.48472076 | -2.424709627 | 0.618014564 | -3.923385901 | 8.73131E-05 | 0.007916022 |
| BTNL8 | 43.32873395 | -0.61504548 | 0.315148221 | -1.951607017 | 0.050984879 | 0.38358267 |
| Symbol | **Base Mean** | **log^2^ FoldChange** | **lfcSE** | **stat** | **P value** | **padj** |
| IKZF3 | 18.36096676 | -0.521750126 | 0.351580903 | -1.484011566 | 0.137805852 | 0.580180635 |
| LOX | 532.9610616 | -0.530829648 | 0.260776039 | -2.035576774 | 0.041792872 | 0.353586632 |
| CD79B | 48.70818608 | -0.839881371 | 0.294047103 | -2.856281736 | 0.004286346 | 0.104659905 |
| CSF3 | 4.372814856 | -0.583248079 | 0.591657889 | -0.985786025 | 0.324238125 | 0.775783892 |
| ARG1 | 409.0026073 | -1.576075947 | 0.438736516 | -3.592306296 | 0.000327764 | 0.019950909 |
| ANPEP | 1206.965593 | -0.719284713 | 0.486509739 | -1.478459021 | 0.139284958 | 0.582279552 |
| CLDN1 | 953.4039935 | -0.712365968 | 0.458682895 | -1.553068526 | 0.120406764 | 0.549062001 |
| LTF | 42.89357794 | -1.129578274 | 0.326884986 | -3.455583228 | 0.000549104 | 0.028058412 |
| ITK | 78.46120379 | -0.577057392 | 0.32384697 | -1.781882943 | 0.074768323 | 0.452387658 |
| SEMA3C | 412.1846013 | -0.449593337 | 0.336675239 | -1.335391751 | 0.181748163 | 0.643789181 |
| MUC5B | 412.1916995 | 1.633602037 | 0.568238534 | 2.874852616 | 0.004042161 | 0.101110501 |
| GBP1 | 684.3729725 | -0.605657852 | 0.300743595 | -2.013867836 | 0.044023411 | 0.359651827 |
| OPRK1 | 76.08689444 | -0.237700812 | 0.558325063 | -0.425739105 | 0.670297964 | 0.922608758 |
| BPIFA1 | 107.8043737 | -1.84963032 | 0.833413796 | -2.219342095 | 0.02646346 | 0.281336506 |
| ADORA1 | 26.88928715 | -1.121745353 | 0.389882292 | -2.877138497 | 0.004012994 | 0.100850535 |
| MS4A1 | 60.54113247 | -1.361789415 | 0.514085721 | -2.648953979 | 0.008074133 | 0.150859901 |
| PLEK | 269.4726533 | -0.693541946 | 0.269845752 | -2.570142165 | 0.010165679 | 0.173082362 |
| TRAT1 | 18.71042191 | -0.616367111 | 0.382109958 | -1.613062151 | 0.106731006 | 0.52454483 |
| IL32 | 949.5778544 | -0.745729714 | 0.365719014 | -2.039078325 | 0.041442211 | 0.353148855 |
| ONECUT1 | 38.66610727 | -1.090125632 | 0.595514084 | -1.830562301 | 0.0671659 | 0.430547043 |
| PYHIN1 | 73.10083182 | -0.314199915 | 0.248098257 | -1.266433381 | 0.205357966 | 0.669029195 |
| FPR2 | 20.09140523 | -0.974477023 | 0.491852926 | -1.981236608 | 0.047564746 | 0.37127565 |
| INAVA | 27.11769252 | -0.635669198 | 0.424527297 | -1.497357653 | 0.134300221 | 0.574341409 |
| CD3G | 14.0692884 | -0.297596004 | 0.405016257 | -0.734775453 | 0.462476259 | 0.851704645 |
| MUC6 | 136.6875155 | -2.738244383 | 0.566956164 | -4.829728572 | 1.36719E-06 | 0.00031252 |
| CCL20 | 25.82572764 | -0.387488113 | 0.58881011 | -0.658086717 | 0.51048241 | 0.870609842 |
| FCRL1 | 13.76456671 | -0.578745256 | 0.460334502 | -1.25722763 | 0.208671221 | 0.672423294 |
| LCK | 66.40913547 | -1.069201208 | 0.308174364 | -3.469468367 | 0.000521489 | 0.027065808 |
| ICOS | 17.30493014 | -0.691910656 | 0.423366241 | -1.634307576 | 0.102194284 | 0.515215526 |
| SP7 | 2.381149278 | -0.861320283 | 0.639541392 | -1.346778009 | 0.178051739 | 0.63859084 |
| TNFRSF17 | 3.221185104 | -0.88088636 | 0.408555386 | -2.156100224 | 0.03107584 | 0.307873926 |
| AOC1 | 712.4411377 | 0.053737927 | 0.549820588 | 0.097737205 | 0.922140972 | 0.984373436 |
| PITX2 | 13.9566229 | -0.66017621 | 0.43904715 | -1.50365675 | 0.132669771 | 0.572229439 |
| FCRL3 | 30.91089859 | -1.076944946 | 0.393296954 | -2.738248883 | 0.006176731 | 0.129104497 |
| LY9 | 30.44716085 | -0.76936259 | 0.35202723 | -2.185520108 | 0.028850733 | 0.296894495 |
| SELL | 139.4593285 | -0.994598104 | 0.320101393 | -3.107134568 | 0.001889104 | 0.061039893 |
| VCAM1 | 688.2915047 | -0.928953877 | 0.283680659 | -3.274646503 | 0.001057943 | 0.04272935 |
| VTCN1 | 18.28184803 | -1.066005834 | 0.497181337 | -2.14409865 | 0.032024983 | 0.312640079 |
| MT1G | 235.4519109 | -0.017069463 | 0.498858245 | -0.034217061 | 0.972704062 | 0.99396982 |
| IKZF1 | 177.1986193 | -0.605653753 | 0.257941782 | -2.348025002 | 0.018873254 | 0.237074642 |
| IL1RAP | 614.5291113 | -1.176782562 | 0.328821363 | -3.578789866 | 0.000345189 | 0.020718082 |
| S100P | 15.22125642 | -0.128276532 | 0.40579294 | -0.316113266 | 0.751916538 | 0.945300587 |
| Symbol | **Base Mean** | **log^2^ FoldChange** | **lfcSE** | **stat** | **P value** | **padj** |
| AZGP1 | 2490.548244 | -0.070062773 | 0.58740699 | -0.119274666 | 0.905057754 | 0.98083868 |
| S100A12 | 8.779529683 | -0.517324928 | 0.47479911 | -1.089565918 | 0.275904403 | 0.742187966 |
| SLAMF6 | 48.89290227 | -0.818685317 | 0.346178185 | -2.364924633 | 0.01803374 | 0.232448099 |
| CXCL10 | 108.3887598 | -1.33933564 | 0.442244207 | -3.02849787 | 0.002457728 | 0.073041775 |
| ANXA1 | 1620.006404 | -0.541852795 | 0.234525832 | -2.31041839 | 0.020865002 | 0.249485975 |
| CFHR1 | 423.3622333 | -0.029074812 | 0.278536768 | -0.104384107 | 0.916864535 | 0.983627064 |
| S100A8 | 115.2203524 | -0.91687509 | 0.43193916 | -2.122694985 | 0.03377942 | 0.320166911 |
| POU2AF1 | 43.13123369 | -1.063896096 | 0.421570058 | -2.523651943 | 0.011614286 | 0.185958388 |
| ZBP1 | 24.53345187 | -0.499482111 | 0.37145438 | -1.344666096 | 0.178733088 | 0.638932505 |
| AQP3 | 6084.457989 | -0.104137418 | 0.396334903 | -0.262751065 | 0.792742455 | 0.956141293 |
| LY75 | 544.4138788 | 0.070407527 | 0.354203963 | 0.19877679 | 0.842437353 | 0.967968846 |
| CRISPLD2 | 1983.480022 | -0.525072887 | 0.254359954 | -2.064290698 | 0.038990162 | 0.342510883 |
| TNFRSF10D | 256.8830901 | 0.052129885 | 0.329566445 | 0.158177161 | 0.8743172 | 0.975261762 |
| IL12B | 19.7627281 | 0.561620856 | 0.532432325 | 1.054821109 | 0.291507149 | 0.75520294 |
| SLAMF7 | 118.4545259 | -1.138811389 | 0.365282515 | -3.117618125 | 0.001823189 | 0.059992121 |
| FLT3 | 22.01867438 | -0.284945601 | 0.285773797 | -0.997101919 | 0.318715042 | 0.773723884 |
| PADI2 | 102.9855786 | -1.164921996 | 0.322944425 | -3.607190294 | 0.000309531 | 0.019277928 |
| PTPN22 | 92.79878047 | -0.777444426 | 0.257720028 | -3.016624012 | 0.002556066 | 0.074219187 |
| GBP5 | 172.5787156 | -1.226779341 | 0.372593171 | -3.292543812 | 0.000992854 | 0.040932015 |
| CD180 | 45.35663959 | -0.882444622 | 0.288585675 | -3.05782545 | 0.002229494 | 0.06804707 |
| ZNF385A | 368.757877 | 0.660783024 | 0.273938584 | 2.412157548 | 0.015858427 | 0.220484462 |
| CD5 | 67.7935608 | -0.217436006 | 0.335960528 | -0.647207001 | 0.517497981 | 0.873143236 |
| CCL17 | 10.40137158 | -0.438390689 | 0.350038714 | -1.252406296 | 0.210421853 | 0.674004841 |
| YAP1 | 1089.290899 | 0.242131209 | 0.257460092 | 0.940461132 | 0.346981078 | 0.790873287 |
| ITM2A | 418.3675483 | -0.687222115 | 0.279319495 | -2.460344258 | 0.01388038 | 0.205546332 |
| IL4R | 1119.233752 | -0.876032115 | 0.284362205 | -3.080691103 | 0.002065208 | 0.064686763 |
| HOXB6 | 125.9311969 | -0.437681511 | 0.369469561 | -1.184621297 | 0.236167213 | 0.704820818 |
| RBP4 | 2520.850964 | 0.050202851 | 0.656137334 | 0.076512718 | 0.939011196 | 0.986458298 |
| IGLL5 | 335.6870996 | -1.223761295 | 0.489910222 | -2.497929705 | 0.012492096 | 0.192311659 |
| BLK | 38.13716104 | -1.489016852 | 0.464687928 | -3.204337278 | 0.001353738 | 0.050371765 |
| IFI44L | 343.9881587 | 0.091737369 | 0.332419879 | 0.27596836 | 0.782572368 | 0.954050069 |
| HOXA5 | 81.69657946 | -0.987667898 | 0.396132175 | -2.493278662 | 0.012656947 | 0.193850477 |
| CCR5 | 63.1096575 | -0.524344151 | 0.277772763 | -1.88767302 | 0.059069861 | 0.409711809 |
| FCGR2B | 149.1211955 | -0.692425332 | 0.326939086 | -2.117903185 | 0.034183268 | 0.321864976 |
| GYPC | 287.5969175 | -0.037033376 | 0.208968445 | -0.177219942 | 0.859335632 | 0.972020509 |
| SFRP2 | 1734.784563 | -1.518348853 | 0.569395199 | -2.666599325 | 0.007662296 | 0.146346451 |
| IL7R | 145.8177301 | -0.422769836 | 0.38044178 | -1.111260272 | 0.266456335 | 0.734678206 |
| SLC7A9 | 319.0913424 | 0.64576607 | 0.417586557 | 1.546424469 | 0.122002088 | 0.55258408 |
| GPR15 | 1.910653785 | -0.084616391 | 0.48682771 | -0.17381178 | 0.862013386 | 0.972594078 |
| COL17A1 | 23.5698525 | -1.386013517 | 0.451408483 | -3.070419736 | 0.002137581 | 0.066181858 |
| PLA2G2D | 12.52857123 | -0.694518838 | 0.623187789 | -1.114461566 | 0.265081224 | 0.733238963 |
| UNC13D | 269.1663048 | -1.088453201 | 0.285631801 | -3.810686331 | 0.000138582 | 0.010867522 |
| Symbol | **Base Mean** | **log^2^ FoldChange** | **lfcSE** | **stat** | **P value** | **padj** |
| IFITM1 | 1324.182334 | -0.439140315 | 0.239943399 | -1.830182938 | 0.067222588 | 0.430781912 |
| SERPING1 | 2640.011991 | -0.307374137 | 0.264685236 | -1.161281762 | 0.245527334 | 0.714271805 |
| APCS | 104.3749273 | 1.18672912 | 0.874874088 | 1.356457045 | 0.174953794 | 0.633917969 |
| SH2D1A | 30.86220557 | -0.900650996 | 0.358192948 | -2.514429729 | 0.011922506 | 0.188366828 |
| S100A14 | 190.8333112 | 0.236189924 | 0.74423882 | 0.317357705 | 0.750972196 | 0.945292301 |
| IFNG | 4.139899985 | -1.97030445 | 0.605655999 | -3.253174168 | 0.001141235 | 0.045326657 |
| CHIA | 8.107688145 | -2.866650597 | 0.974795387 | -2.940771608 | 0.003273958 | 0.087934428 |
| KRT6A | 40.6204229 | -0.508796536 | 0.682957839 | -0.744989671 | 0.456277964 | 0.848734486 |
| IRF8 | 442.5204834 | -1.242876186 | 0.329429175 | -3.772817593 | 0.000161414 | 0.012126955 |
| ICAM1 | 770.1427874 | -0.654045592 | 0.281307801 | -2.325017613 | 0.020071018 | 0.244083779 |
| CD3E | 82.55342376 | -0.744949886 | 0.327728927 | -2.273067233 | 0.023022129 | 0.262010847 |
| CLEC2B | 253.3494018 | -0.457947084 | 0.209699865 | -2.183821549 | 0.028975364 | 0.297435342 |
| CD8B | 48.23089723 | 0.195642493 | 0.251815996 | 0.776926394 | 0.437202198 | 0.842223743 |
| APOA2 | 406.3013919 | 3.005577321 | 0.972740821 | 3.089802808 | 0.002002894 | 0.063601512 |
| CCR2 | 43.50794246 | -0.572174989 | 0.331623251 | -1.725376576 | 0.08445963 | 0.475454398 |
| PLA2G1B | 154.2883507 | -2.606773153 | 0.493474123 | -5.282492093 | 1.27438E-07 | 4.72115E-05 |
| IFITM2 | 1654.765588 | -0.09774092 | 0.237606906 | -0.411355553 | 0.68081184 | 0.925576254 |
| TDGF1 | 7.976958786 | -0.897811101 | 0.448857426 | -2.00021443 | 0.045477114 | 0.363935476 |
| TNFRSF13B | 3.4008417 | -0.792071725 | 0.656915584 | -1.205743546 | 0.227916389 | 0.697392342 |
| FASLG | 11.4203446 | -0.21438593 | 0.372008388 | -0.576293268 | 0.564416973 | 0.887217486 |
| LYZ | 9611.77592 | -0.619589889 | 0.477246443 | -1.298259837 | 0.194198063 | 0.657537626 |
| IL20 | 2.642924869 | 0.466420606 | 0.779156768 | 0.598622286 | 0.549424791 | 0.881269818 |
| JCHAIN | 584.0525328 | -1.433083446 | 0.459609417 | -3.118046308 | 0.001820542 | 0.059992121 |
| CD3D | 37.66111606 | -1.125459717 | 0.35700603 | -3.152494983 | 0.001618816 | 0.055476077 |
| CCL21 | 536.3046253 | -1.149231745 | 0.484063852 | -2.374132546 | 0.017590235 | 0.229345494 |
| HLA-DQA1 | 760.8544128 | -0.124524286 | 0.289751587 | -0.429762224 | 0.667368615 | 0.921481682 |
| UBASH3A | 31.23016096 | -0.581015705 | 0.331100391 | -1.75480223 | 0.079293142 | 0.465137654 |
| MST1R | 155.6795633 | -0.875518674 | 0.455995169 | -1.920017433 | 0.054855697 | 0.395664441 |
| PLA2G10 | 71.9800018 | -0.519618711 | 0.292156231 | -1.778564532 | 0.075311183 | 0.454014309 |
| CFHR4 | 2250.224391 | -0.1580159 | 0.253579715 | -0.623140932 | 0.533191914 | 0.877504186 |
| C8A | 265.0181477 | 0.288875341 | 0.628690055 | 0.45948769 | 0.64588399 | 0.916244139 |

Table S5. Differential gene expression analysis of 150 immune gene in SHPCI-PanNET cohort

| GeneName | pvalue | FoldChange | GeneName | P value | FoldChange |
| --- | --- | --- | --- | --- | --- |
| IL16 | 1.27E-19 | 2.456140351 | FASLG | 0.573698487 | -0.052334944 |
| IRF4 | 1.50E-15 | 2.313645621 | CCR2 | 0.352591811 | -0.052653229 |
| LRG1 | 1.79E-12 | 2.039688716 | JCHAIN | 0.652363737 | -0.059729527 |
| MUC1 | 7.92E-15 | 1.865782493 | TNFRSF13B | 0.558170935 | -0.062454611 |
| CXCL9 | 5.13E-16 | 1.726653696 | CD8B | 0.347769555 | -0.070599384 |
| CCL19 | 1.39E-17 | 1.616044616 | LYZ | 0.6068733 | -0.107061503 |
| CR2 | 2.17E-10 | 1.599056604 | TDGF1 | 0.465885132 | -0.115845539 |
| PIGR | 3.92E-24 | 1.546816479 | SLC7A9 | 0.065196437 | -0.14244186 |
| CD79A | 2.05E-12 | 1.406867357 | SH2D1A | 0.142826 | -0.158536585 |
| TCF21 | 5.05E-12 | 1.255294571 | PLA2G1B | 0.393671779 | -0.187609585 |
| CCR4 | 7.20E-26 | 1.195454545 | APCS | 0.125961571 | -0.191225166 |
| CSF3 | 8.72E-10 | 1.151204511 | GYPC | 0.053915957 | -0.222745626 |
| IL2RG | 1.28E-11 | 1.14959115 | SELL | 2.14E-05 | -0.223105902 |
| CRTAM | 3.65E-12 | 1.020573566 | KRT6A | 0.249448681 | -0.227562247 |
| LGALS3 | 3.98E-14 | 0.972660996 | ICAM1 | 0.279272907 | -0.235382309 |
| SIT1 | 8.77E-14 | 0.938983051 | ICOS | 3.42E-06 | -0.235575391 |
| ONECUT1 | 3.89E-07 | 0.93729057 | LCK | 3.26E-06 | -0.235797888 |
| LTF | 2.39E-09 | 0.914037855 | MT1G | 9.19E-05 | -0.243474715 |
| MUC6 | 1.44E-06 | 0.882604055 | HOXA5 | 0.027830813 | -0.250705219 |
| SEMA3C | 3.64E-09 | 0.87456979 | CCR5 | 0.038973103 | -0.262964325 |
| BTNL8 | 4.96E-10 | 0.836029179 | CD5 | 0.004613859 | -0.284706637 |
| TNFRSF1B | 1.36E-12 | 0.833231147 | FCGR2B | 0.04077409 | -0.295576685 |
| PYHIN1 | 5.03E-07 | 0.787998248 | SP7 | 5.20E-06 | -0.3185149 |
| ANPEP | 1.11E-09 | 0.697782191 | GPR15 | 0.071422304 | -0.328968903 |
| PITX2 | 9.87E-06 | 0.675675676 | SLAMF7 | 0.000911646 | -0.333657903 |
| OPRK1 | 1.57E-08 | 0.651467655 | IL7R | 0.060806085 | -0.354493581 |
| CLDN1 | 2.34E-09 | 0.586706949 | S100A8 | 0.000281942 | -0.383005311 |
| ADORA1 | 6.19E-08 | 0.576142132 | IL12B | 0.000901057 | -0.40030349 |
| FPR2 | 5.73E-07 | 0.556637907 | RBP4 | 0.011940381 | -0.408542247 |
| FCRL3 | 1.28E-05 | 0.555222763 | S100A12 | 0.000153207 | -0.413931145 |
| ARG1 | 9.94E-10 | 0.518426843 | CRISPLD2 | 0.000794936 | -0.417349727 |
| ITK | 3.13E-09 | 0.510837229 | CD180 | 0.003945666 | -0.441776167 |
| CD3G | 9.18E-07 | 0.5 | CXCL11 | 3.90E-14 | -0.460482251 |
| YAP1 | 0.006115776 | 0.481375358 | IKZF3 | 5.57E-10 | -0.46072342 |
| POU2AF1 | 0.000303825 | 0.472805139 | PTPN22 | 0.00202921 | -0.460965084 |
| TRAT1 | 1.14E-07 | 0.465895954 | IL1RAP | 0.000108977 | -0.483024691 |
| IFI44L | 0.019183968 | 0.454907823 | TNFRSF10D | 0.000896845 | -0.502879079 |
| BPIFA1 | 5.37E-08 | 0.441581186 | PADI2 | 0.001763563 | -0.513829787 |
| SLAMF6 | 0.000154143 | 0.437600644 | ZBP1 | 0.000482334 | -0.537652503 |
| CD79B | 6.62E-10 | 0.426246905 | CTLA4 | 5.59E-16 | -0.601760176 |
| AOC1 | 8.02E-06 | 0.411272988 | CCL20 | 2.44E-06 | -0.627855564 |
| GeneName | pvalue | FoldChange | GeneName | P value | FoldChange |
| BLK | 0.014012012 | 0.394894895 | AZGP1 | 0.000150825 | -0.629545455 |
| CXCL10 | 0.000222914 | 0.390402844 | TNFRSF17 | 7.21E-06 | -0.640296181 |
| GBP5 | 0.002962802 | 0.369874137 | CNR1 | 2.12E-13 | -0.642288557 |
| FLT3 | 0.001694032 | 0.35997578 | AQP3 | 0.000580949 | -0.65857438 |
| LY75 | 0.000692703 | 0.340594059 | S100P | 0.000150553 | -0.708077994 |
| FCRL1 | 3.07E-06 | 0.337926033 | IKZF1 | 0.000107577 | -0.74241557 |
| ITM2A | 0.006172291 | 0.328582146 | INAVA | 7.77E-07 | -0.812216052 |
| PLA2G2D | 0.080789207 | 0.309485095 | SELE | 1.81E-17 | -0.840054372 |
| COL17A1 | 0.075745111 | 0.273179557 | CCL17 | 0.006005909 | -0.884250474 |
| SFRP2 | 0.058651324 | 0.266429137 | IL6 | 5.97E-19 | -0.891509434 |
| S100A14 | 0.224362376 | 0.263092269 | TIGIT | 9.51E-19 | -0.919744642 |
| ZNF385A | 0.004545525 | 0.260312945 | IGLL5 | 0.013042573 | -0.963428571 |
| IL4R | 0.007688941 | 0.247569185 | CCL5 | 8.62E-11 | -0.967517401 |
| LY9 | 1.68E-05 | 0.211232449 | CFHR1 | 0.00028082 | -1.069508804 |
| UNC13D | 0.106557016 | 0.177118079 | MS4A1 | 7.84E-08 | -1.089447236 |
| CHIA | 0.24219023 | 0.177065767 | ANXA1 | 0.000254055 | -1.095019157 |
| CD3E | 0.33593915 | 0.136874362 | THEMIS | 8.53E-17 | -1.24071618 |
| SERPING1 | 0.125821408 | 0.134620767 | VCAM1 | 3.67E-05 | -1.507344633 |
| IFITM1 | 0.120283052 | 0.115075107 | SIRPG | 6.81E-14 | -1.656395892 |
| IFNG | 0.238073119 | 0.114191251 | MZB1 | 3.23E-24 | -1.698795181 |
| HOXB6 | 0.009915356 | 0.112924113 | IL32 | 3.22E-07 | -1.853768279 |
| CLEC2B | 0.344929731 | 0.099445599 | PLEK | 9.47E-08 | -1.902972028 |
| IRF8 | 0.271584908 | 0.08443143 | GBP1 | 1.40E-08 | -1.903979239 |
| IL20 | 0.628343037 | 0.08310992 | VTCN1 | 5.27E-05 | -1.915017462 |
| APOA2 | 0.348379391 | 0.072125813 | LOX | 5.68E-10 | -2.053858521 |
| IFITM2 | 0.439713522 | 0.068045203 | MYO10 | 8.63E-13 | -2.076056338 |
| CD3D | 0.756739039 | 0.046738072 | MUC5B | 1.29E-08 | -2.246282528 |
| HLA-DQA1 | 0.786740807 | 0.02310803 | CD70 | 5.28E-24 | -2.29787234 |
| CCL21 | 0.777630101 | 0.0205 | AIM2 | 6.71E-13 | -2.459622642 |
| PLA2G10 | 0.868833505 | 0.014240046 | HOXA3 | 1.58E-22 | -3.187265918 |
| CFHR4 | 0.900895195 | 0.013788349 | TRIM29 | 2.16E-27 | -3.216580311 |
| UBASH3A | 0.859561641 | 0.00786557 | REG1B | 1.69E-14 | -3.434254144 |
| C8A | 0.930226079 | 0.006158759 | ZAP70 | 4.36E-15 | -3.552359033 |
| MST1R | 0.862084365 | -0.018139066 | LAX1 | 7.34E-27 | -3.649278579 |

Table S6. Predictive accuracy of nomogram with individual predictor in training and validation cohort

|  | Training cohort | | Validation cohort | |  |
| --- | --- | --- | --- | --- | --- |
|  | C-index | 95% CI | C-index | 95% CI | |
| Nomogram | 0.917 | 0.884-0.950 | 0.864 | 0.798-0.930 | |
| ISpnet | 0.796 | 9.714-0.878 | 0.714 | 0.607-0.821 | |
| Liver metastasis | 0.725 | 0.633-0.817 | 0.673 | 0.564-0.782 | |
| WHO grade | 0.695 | 0.631-0.759 | 0.676 | 0.575-0.777 | |
