# Supplementary figures and images for: From the Immune Profile to the Immunoscore: Signatures for Improving Postsurgical Prognostic Prediction of Pancreatic Neuroendocrine Tumors
Source: Front Immunol. 2021 Apr 23;12:654660. doi: 10.3389/fimmu.2021.654660 (PMC8102869; doi:10.3389/fimmu.2021.654660)

# Figure S1

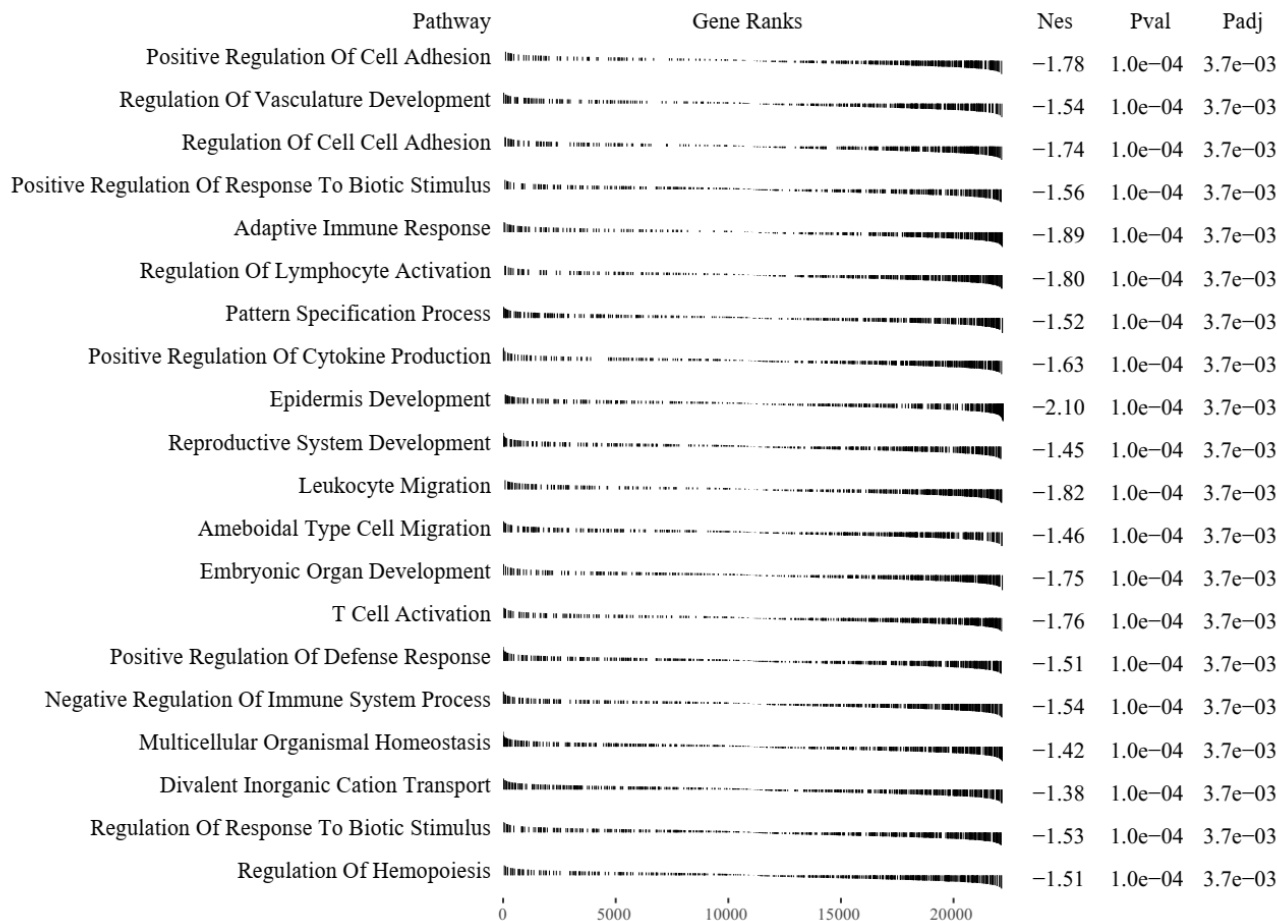

Supplement: Supplementary file 2 [file Image_1.pdf]

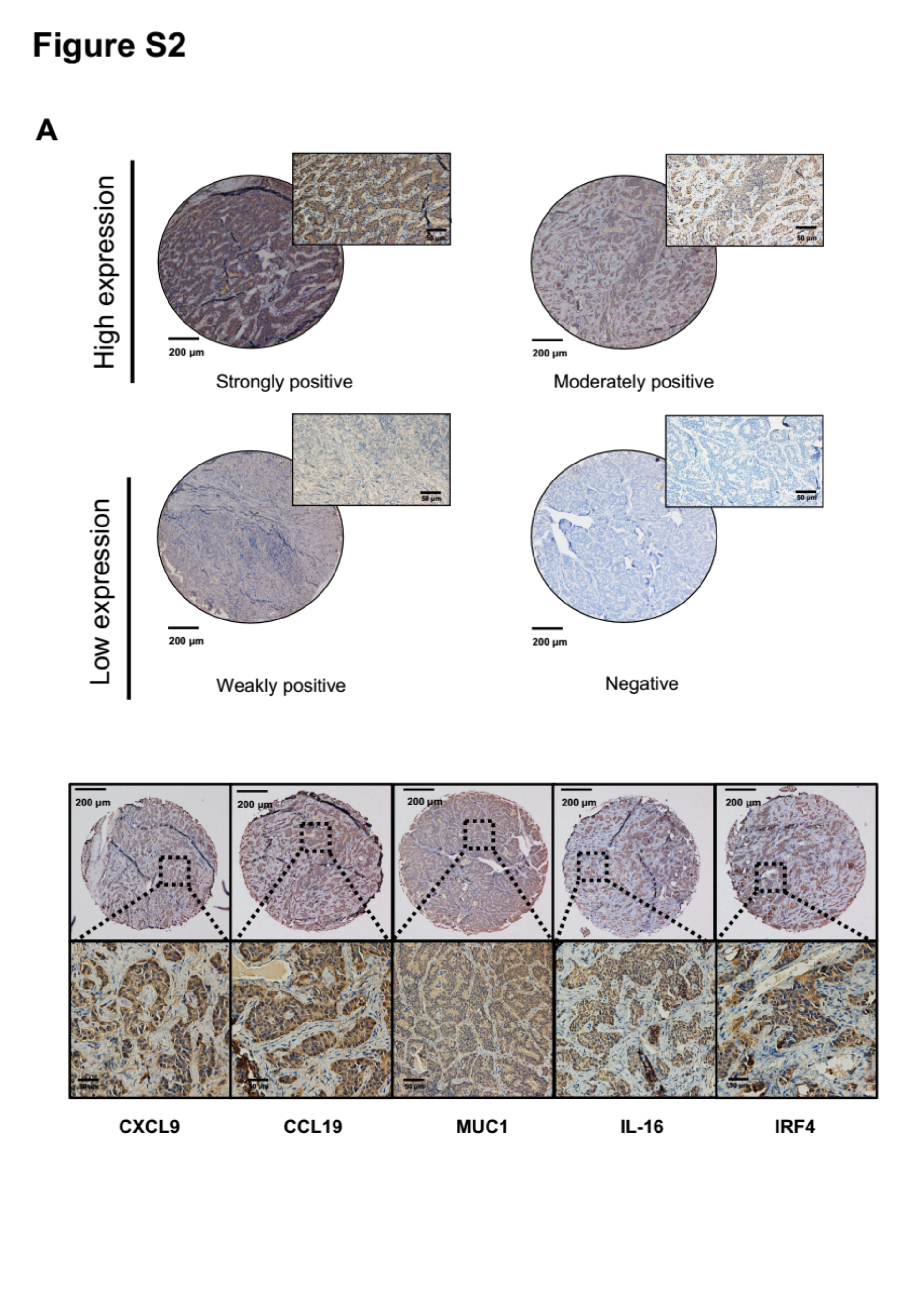

Supplement: Supplementary file 3 [file Image_2.tif]

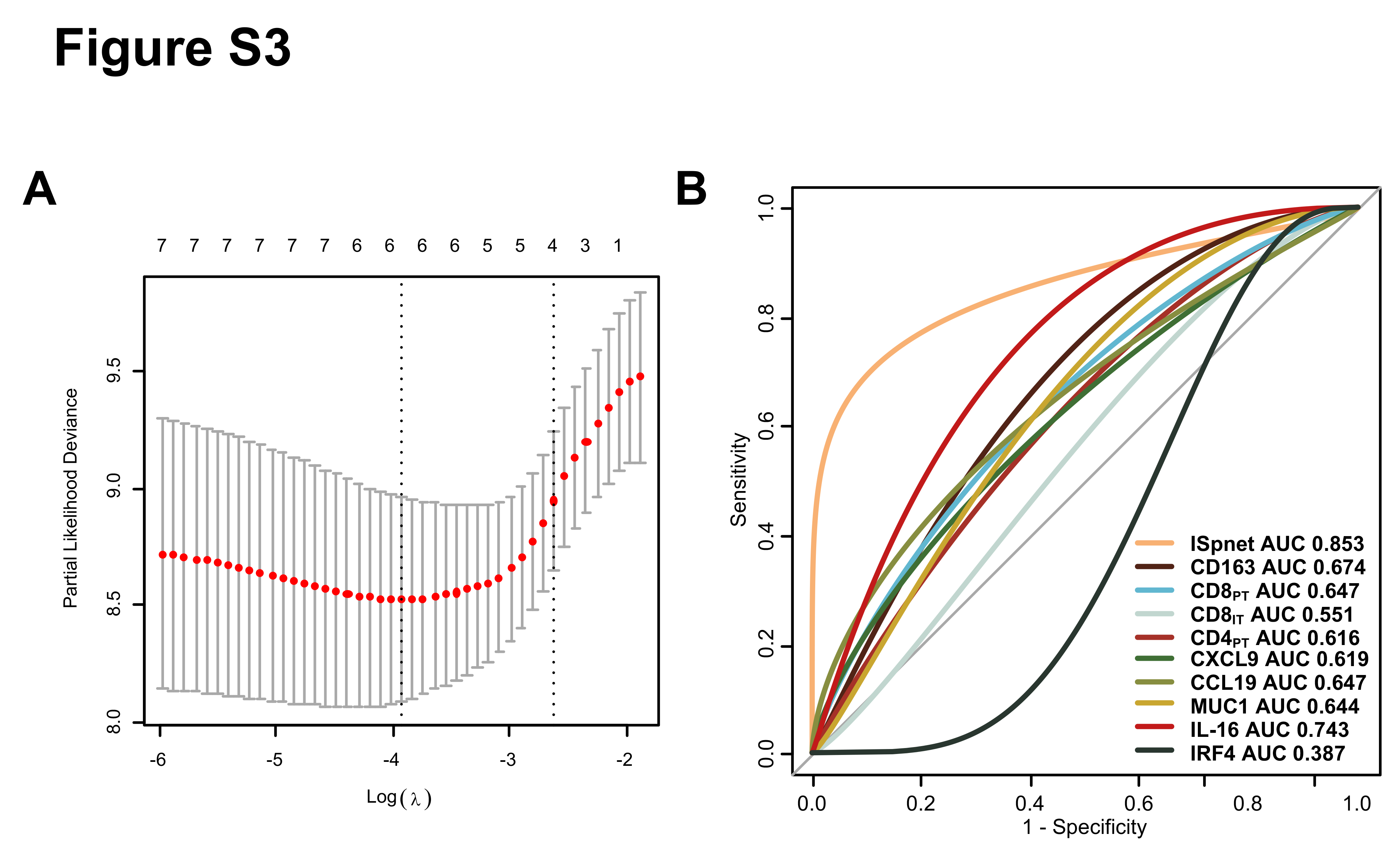

Supplement: Supplementary file 4 [file Image_3.jpg]
